# Natural Products as Novel Therapeutic Agents for Triple-Negative Breast Cancer: Current Evidence, Mechanisms, Challenges, and Opportunities

**DOI:** 10.3390/molecules30061201

**Published:** 2025-03-07

**Authors:** Qingzhou Li, Zhen Ye, Guilin Wang, Yuhui Chen, Jinghong Deng, Dong Wang, Yumei Wang

**Affiliations:** 1 State Key Laboratory of Southwestern Chinese Medicine Resources, School of Pharmacy, Chengdu University of Traditional Chinese Medicine, Chengdu 611137, China; liqingzhou@stu.cdutcm.edu.cn; 2 School of Basic Medical Sciences, Chengdu University of Traditional Chinese Medicine, Chengdu 611137, China; yezhen@stu.cdutcm.edu.cn (Z.Y.); guilinwang@stu.cdutcm.edu.cn (G.W.); yhchen@cdutcm.edu.cn (Y.C.); wddengjinghong@163.com (J.D.)

**Keywords:** triple-negative breast cancer, herbal medicine, natural products, therapeutic strategies, mechanism

## Abstract

Breast cancer (BC) tops the list of causes for female fatalities globally, with the elusive triple-negative breast cancer (TNBC) constituting 10–20% of all cases. Current clinical strategies for combating TNBC encompass a multifaceted approach, including surgical intervention, radiation therapy, chemotherapy, and advanced targeted drugs and immunotherapies. While these modalities have catalyzed significant advancements in TNBC management, lingering limitations continue to pose formidable challenges. There is an acute need for novel therapeutics in the realm of TNBC treatment. Natural products (NPs) have emerged as a rich reservoir for pharmaceutical innovation, owing to their extraordinary range of structures and physicochemical properties. Scholars have reported diverse evidence of NPs’ efficacy against TNBC. This review aims to comprehensively explore the bioactive constituents, specifics and commonalities of chemical structure, and pharmacological mechanisms of NPs, specifically examining their multifaceted roles in impeding TNBC. NPs, which have recently garnered significant interest, are intriguing in terms of their capacity to combat TNBC through multifaceted mechanisms, including the suppression of tumor cell proliferation, the induction of apoptosis, and the inhibition of tumor metastasis. These natural agents primarily encompass a range of compounds, including terpenoids, glycosides, phenolic compounds, and alkaloids. An in-depth exploration has unveiled their involvement in key signaling pathways, including the transforming growth factor-beta (TGF-β), vascular endothelial growth factor A (VEGFA), phosphoinositide 3-kinase/protein kinase B (PI3K/AKT), Wingless/Int-1 (Wnt) /β-catenin, and mitogen-activated protein kinase (MAPK) pathways. Meanwhile, this review also looks at the challenges and opportunities that arise from harnessing natural compounds to influence TNBC, while outlining the prospective trajectory for future research in the field of NPs.

## 1. Introduction

Breast cancer (BC), which accounted for 670,000 deaths globally in 2022, has been defined as a highly life-threatening disease among the female population by the World Health Organization (WHO) [1]. The complexity of BC is strongly related to its heterogeneity, which results in diverse clinical and therapeutic outcomes. Among the various subtypes, TNBC is particularly aggressive. Unlike luminal A/B BC, human epidermal growth receptor 2 (HER2)-positive BC, and basal-like BC, TNBC lacks expression of estrogen receptors (ERs), progesterone receptors (PRs), and HER2, making it unresponsive to hormone therapy, endocrine therapy, and HER2-targeted therapy [2]. TNBC treatment currently relies primarily on chemotherapy as a therapeutic modality [3]. For instance, paclitaxel- and anthracycline-based chemotherapy drugs are currently the principal choices for treating TNBC, with platinum-based compounds demonstrating promising effects in certain treatment contexts [4,5,6,7]. However, the induction of endogenous or acquired resistance in early-stage treatment, significant adverse effects, and potential non-specific off-target effects severely limit the utility of these chemotherapy drugs [8]. Consequently, novel therapeutic strategies, such as targeted therapy and immunotherapy, are being continuously developed to improve their treatment efficacy and survival rates [9,10]. For example, poly adenosinediphosphate-ribose polymerase (PARP) inhibitors have shown therapeutic potential in patients with breast cancer gene (BRCA) mutations. Moreover, other synthetic small-molecule drugs, such as tyrosine kinase inhibitors, including Lapatinib and Afatinib, have also demonstrated commendable efficacy by targeting multiple tyrosine kinase receptors, thereby disrupting the signal transduction pathways of cancer cells [11]. In addition, immune checkpoint inhibitors, such as pembrolizumab and atezolizumab, have displayed breakthrough activity in certain TNBC patients.

Traditional Chinese medicines (TCMs) are regarded as valuable complementary and alternative therapies with significant anticancer potential [12,13]. Herbal medicines (HMs), which have served as the primary remedies in many ancient civilizations, have garnered significant interest in cancer drug discovery. Notably, numerous NPs derived from HMs have demonstrated antitumor effects, a discovery that gained considerable attention following the identification of paclitaxel’s remarkable anticancer activity. As a result, HMs-associated NPs have become a focal point in drug discovery, with research in this area experiencing substantial growth. Recently, it was found that approximately 30% of drugs approved by the U.S. Food and Drug Administration (FDA) are HMs extracts and HM-associated NPs [14]. Paclitaxel, for instance, originally extracted from the herbal plant Taxus chinensis, remains a first-line treatment for various carcinomas. However, many NPs with potential therapeutic benefits are yet to be fully explored. HMs are recognized as a rich source of medicinal compounds, providing advantages such as cost-effectiveness and a wide array of structural and functional variations. The therapeutic efficacy of HMs and associated NPs in treating TNBC has been demonstrated through their ability to target multiple signaling pathways and exhibit various bioactivities.

In this review, we present a comprehensive analysis of the chemical characteristics, bioactivities, and botanical origins of NPs identified as potential adjuvant treatment for TNBC. The primary compounds within these NPs include terpenoids, glycosides, phenolics, and alkaloids. These NPs have been shown to inhibit TNBC through multiple mechanisms, such as the suppression of angiogenesis, prevention of metastasis, induction of apoptosis, interruption of the cell cycle, and regulation of autophagy. The key pathways involved include TGF-β, VEGFA, PI3K/AKT, Wnt/β-catenin, and MAPK. Consequently, NPs offer significant potential for the development of innovative and effective therapies for TNBC.

## 2. Activity of Natural Products in Treatment of Triple-Negative Breast Cancer

### 2.1. Terpenoids

Terpenoids, which represent the largest class of compounds produced by plants, are derived from mevalonic acid, and are characterized by a molecular framework of isoprene units [15]. The compounds are connected in various ways to form polymers: monoterpenes consist of two isoprene units, sesquiterpenes consist of three, diterpenes consist of four, etc. Terpenoids hold significant importance in the fields of biology, pharmacology, and chemistry. Among their numerous biological properties, their antitumor effects are particularly noteworthy, encompassing diverse activities such as anti-proliferative, apoptotic, anti-angiogenic, and anti-metastatic actions [1,16].

**Figure 1:**
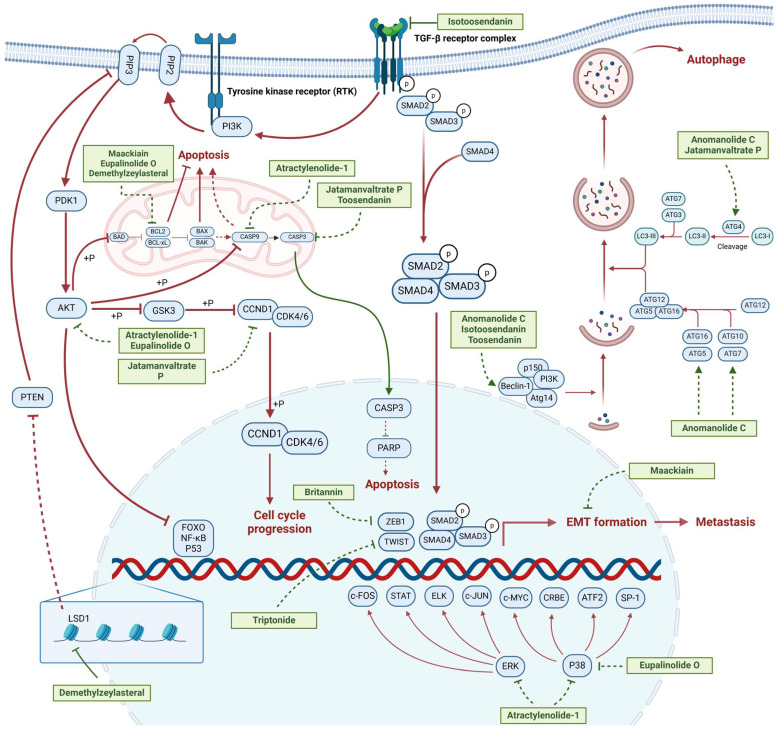
The regulation of terpenoids in TNBC.

Terpenoids undergo enzymatic elongation and cyclization reactions, resulting in compounds with high hydrophobicity and structural complexity. Compared to non-terpenoid NPs, terpenoids exhibit more intricate ring structures and chiral stereochemistry. In proteins, the number of hydrophobic amino acids correlates with protein folding; thus, hydrophobic residues are prevalent in protein active pockets and interfaces that are crucial for ligand binding. The hydrophobic effect is considered a significant driving force for molecular recognition in protein–ligand interactions. Consequently, hydrophobic terpenoids may demonstrate favorable properties in protein–ligand binding. Some terpenoid compounds have been found to possess inhibitory activity against protein–protein interactions, targeting various drug sites, highlighting their broad applicability in drug discovery endeavors [17,18]. Terpenoids are widely distributed in nature and exhibit diverse biological activities, including anti-TNBC effects (Figure 1, Table 1 and Table S1).

#### 2.1.1. Anti-Metastasis

Terpenoids have been reported to induce apoptosis in TNBC cells, which is a form of programmed cell death crucial for the development, homeostasis, and defense mechanisms of multicellular organisms. Appropriate apoptosis function can prevent tumorigenesis [19]. Terpenoids such as atractylenolide-1 (AT-1), demethylzeylasteral (T-96), isotoosendanin (ITSN), toosendanin (TSN), Eupalinolide O (EO), and Jatamanvaltrate P have been documented to modulate the expression of apoptosis-related proteins, including B-cell lymphoma 2 (Bcl2), Bcl-2-associated X protein (Bax), and Caspase-3, thereby inducing death of TNBC cells [20,21,22,23,24]. Apoptosis is regulated by multiple signaling pathways. T-96 significantly reduces lysine-specific demethylase 1 (LSD1) protein levels, increases its target protein phosphatase and tensin homolog (PTEN), and enhances histone methylation. This action ultimately downregulates the PI3K/AKT signaling pathway, effectively inhibiting the growth of TNBC cells and promoting apoptosis [25]. Maackiain (MA) regulates mitochondrial apoptosis signaling in TNBC by promoting GADD45A through the inhibition of microRNA(miR)-374a [26]. EO may induce apoptosis in TNBC cells by modulating reactive oxygen species (ROS) production and the AKT/p38 MAPK pathway [23]. Terpene compounds such as Cycloart-23E-ene-3β, 25-diol, germacrone, and β-Bisabolene have also been reported to induce apoptosis in TNBC cells [27,28,29].

#### 2.1.2. Regulation of Cell Cycle

Disruptions in the cell cycle, which is essential for regulated cell proliferation, are pivotal in tumor development and progression, and research into targeting these disruptions offers promising strategies for cancer treatment. Terpenoids, such as AT-1, Jatamanvaltrate P, Cucurbitacin B, Lineariifolianoid E, and germacrone, have been shown to inhibit the cell cycle, effectively suppressing the growth and proliferation of TNBC cells [21,24,27,29,30,31,32]. They regulate the expression of cell cycle proteins, such as cyclin-dependent kinase (CDK1) and Cyclin D1 (CCND1), leading to cell cycle arrest and inhibition of TNBC cell growth [28,32]. The progression of the cell cycle also depends on the regulation of intracellular signaling pathways. Cucurbitacin B may inhibit the proliferation of human BC cells by disrupting the microtubule network, downregulating Cellular myelocytomatosis oncogene (c-Myc) and nucleophosmin/B23, and interfering with the trafficking of nucleophosmin/B23 from the nucleolus to the nucleoplasm, leading to G2/M arrest [33,34].

#### 2.1.3. Modulation of Autophagy

Autophagy is a self-degradative process that is crucial for regulating metabolism, maintaining energy balance, and controlling cell development and death in response to stress or starvation [35]. In cancer cells, autophagy has a dual role, both promoting tumor cell survival and growth, and potentially inhibiting tumor initiation and progression [36]. As research advances, a better understanding of the complex mechanisms of autophagy may offer new directions for cancer treatment. Terpenoid compounds such as Anomanolide C (AC), ITSN, TSN, and Jatamanvaltrate P have been reported to modulate autophagy. Treatment with these compounds increases the LC3B-II/LC3B-I ratio in TNBC cells, leading to autophagy activation and subsequent inhibition of TNBC [20,24,37,38,39].

Terpenoid compounds show significant potential in inhibiting TNBC by targeting multiple mechanisms, including metastasis suppression and apoptosis induction, demonstrating their multifaceted abilities to hinder the progression of TNBC. For instance, AC has been demonstrated to concurrently impede cell metastasis and modulate autophagy. AT-1 exhibits the potential to inhibit metastasis, arrest the cell cycle, and provoke apoptosis. Similarly, Jatamanvaltrate P has been noted for its ability to halt the cell cycle, influence autophagy, and induce apoptosis. Collectively, these active terpenoid compounds present substantial potential as therapeutic agents for TNBC. Nonetheless, a more profound understanding of their underlying mechanisms is essential, necessitating further research. Additionally, extensive preclinical studies will be pivotal in advancing their translation into clinical practice.

**Table 1:** Profiles of terpenoids in NPs with therapeutic effects on TNBC.

Natural Products	Structure	Sources	Concentration In Vitro	Dosage In Vivo	Biological Activities/Mechanisms	Potential Targets	Reference
Anomanolide C	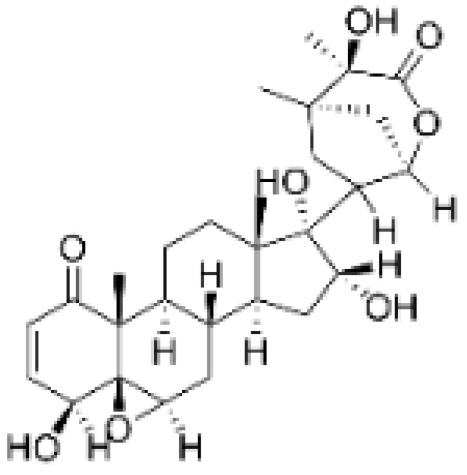	*Tubocapsicum anomalum (S. Vidal)* M. J. Pires	0.25, 1, 2 μM	25, 50 mg/kg	Induces glutathione peroxidase 4 (GPX4) ubiquitination to trigger autophagy-dependent ferroptosis in TNBC.	GPX4	[38]
Atractylenolide-1	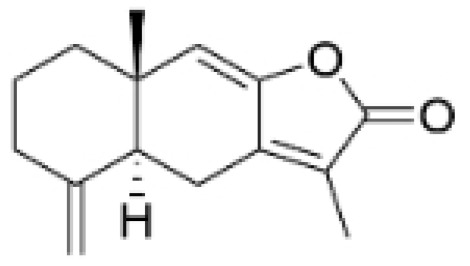	*Atractylodes lancea*	25, 50 μM	20, 50 mg/kg	Inhibits phosphorylation of the Extracellular signal-regulated kinase (ERK) family; suppresses the expression of Triosephosphate Isomerase 1 (TPI1) and Glucose-6-phosphate isomerase GPI (GPI), thereby affecting the glycolytic/gluconeogenic pathway.	TPI1 and GPI	[21]
Britannin	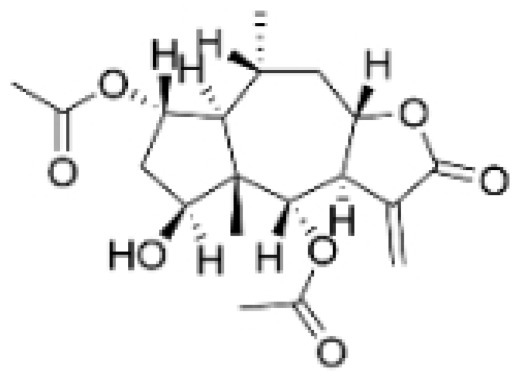	*Inula japonica* Thunb	5, 10, 20 μM	5, 10 mg/kg	Induces Zinc finger e-box binding homeobox 1 (ZEB1) degradation, inhibiting invasion and metastasis of TNBC cells.	ZEB1	[40]
Demethylzeylasteral	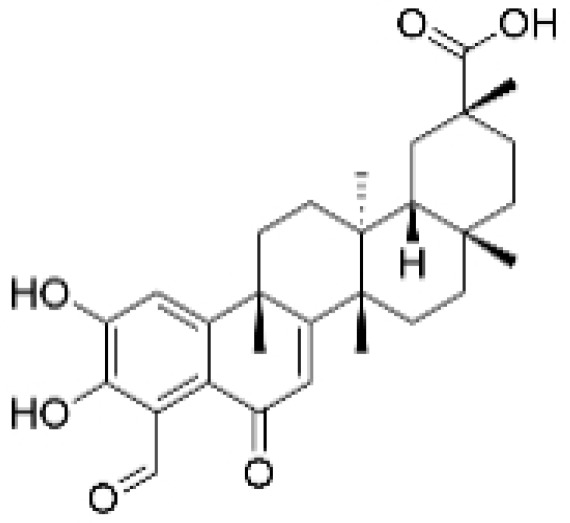	*Tripterygium wilfordii*	5, 10 μM	4 mg/kg	Reduces the expression of LSD1 protein, enhances the expression of its target protein PTEN, and suppresses the PI3K/AKT signaling pathway.	LSD1	[25]
Isotoosendanin	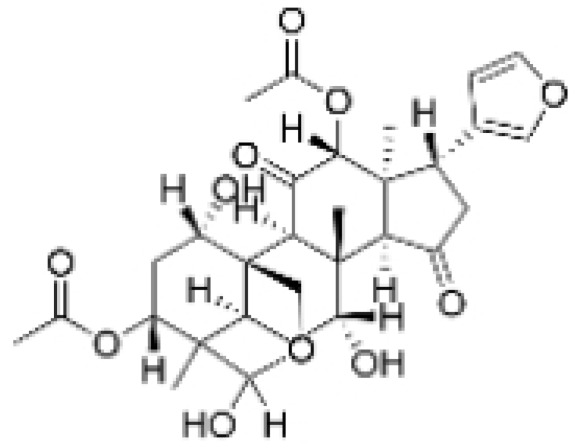	*Melia azedarach* L.	0.1, 0.3, 1 μM	0.1, 1 mg/kg	Directly engages with the kinase domain of TGF-β receptor type-1 (TGFβR1), thereby abolishing TGF-β-induced epithelial–mesenchymal transition (EMT) in the TNBC microenvironment.	TGFβR1	[20,37]
Toosendanin	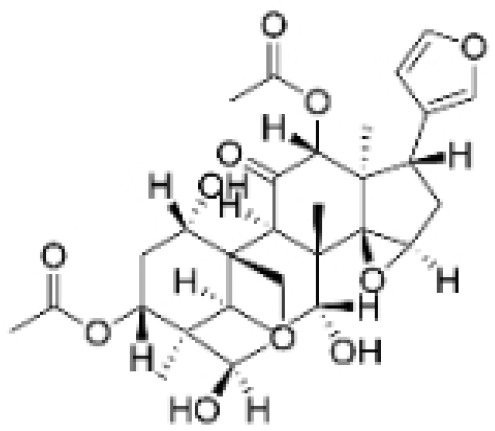	*Melia azedarach* L.	0.1, 1, 5 μM	/	Inhibits autophagy by alkalizing lysosomal pH.	/	[20,39]
Jatamanvaltrate P	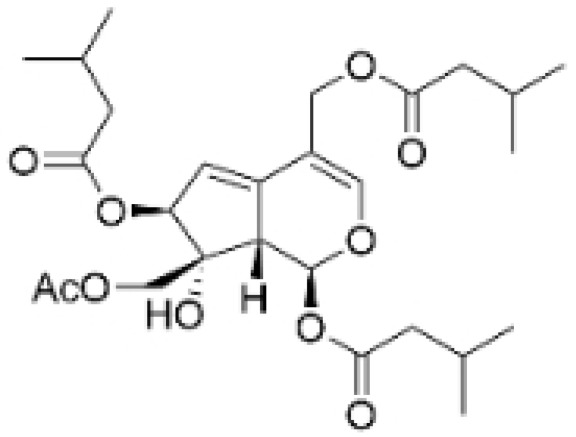	*Valeriana jatamansi* A. DC.	2.5, 5 μM	15 mg/kg	Enhances PARP and caspase cleavage, indicating apoptosis induction, and reducing Cyclin B1, CCND1, and Cell division cycle 2 (Cdc-2) expression, which are crucial for cell cycle progression.	/	[24]
Maackiain	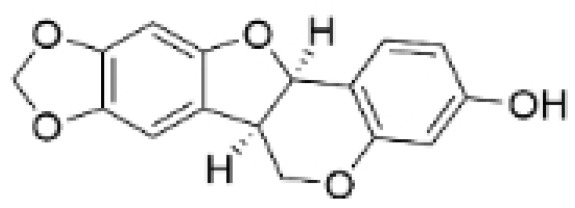	*Spatholobus suberectus* H. S. Lo	1, 2, 2.5, 5 μM	25, 50 mg/kg	Enhances the induction of GADD45α protein and mRNA through the inhibition of miR-374a.	/	[26]
Triptonide	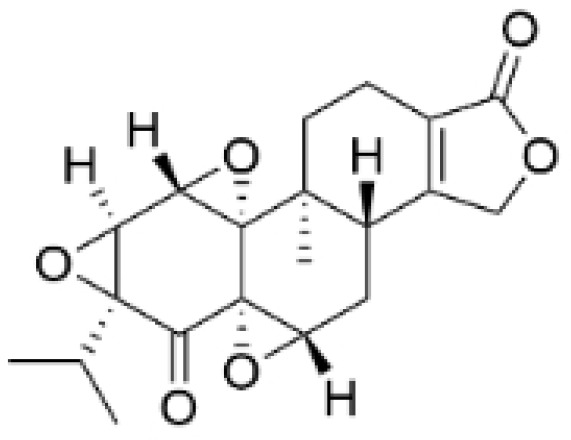	*Tripterygium wilfordii* Hook. f.	0.2 μM	5 mg/kg	Suppresses the tumorigenesis and metastasis of TNBC via triggering the degradation of Twist1 and Notch1 oncoproteins.	/	[41,42]
Eupalinolide O	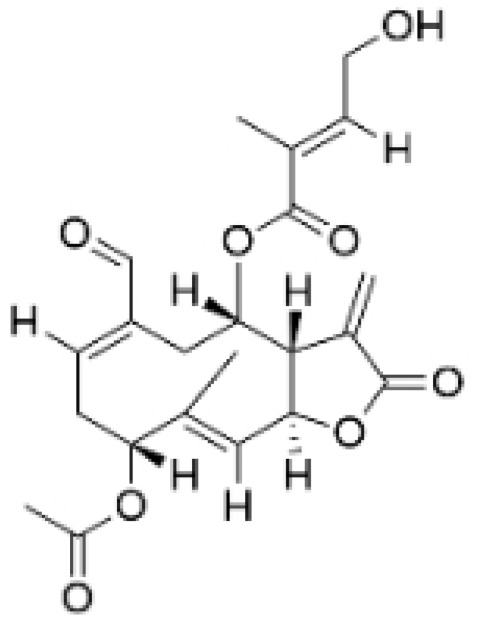	*Eupatorium lindleyanum* DC.	5, 10 μM	15, 30 mg/kg	Induces apoptosis, which is orchestrated through ROS generation and the AKT/p38 MAPK signaling pathway.	/	[23]

### 2.2. Glycosides

Glycosides are characterized by the presence of a sugar moiety linked to a non-sugar portion known as the aglycone. These compounds display distinct biological activities due to their varied aglycone structures, while their shared sugar moieties contribute to similar functionalities.

**Figure 2:**
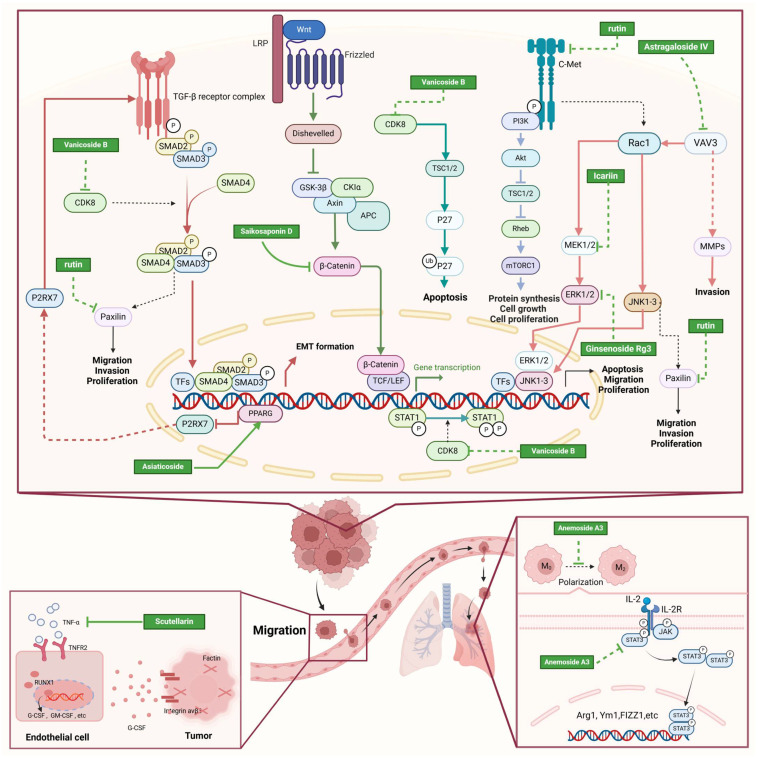
The regulation of glycosides in TNBC.

NPs contain numerous glycoside components, and research has documented their inhibitory effects on TNBC (Figure 2, Table 2 and Table S1).

#### 2.2.1. Anti-Metastasis

Recent studies have highlighted that glycoside compounds, like icariin, Vanicoside B, asiaticoside (AS), Astragaloside IV (AS-IV), scutellarin (SC), Platycodin D (PD), and rutin, have the potential to inhibit the metastasis of TNBC cells through various mechanisms [43,44,45,46,47,48,49,50,51,52,53]. Icariin, for instance, has been reported to reduce TNBC cell invasion by targeting and suppressing the c-Jun N-terminal kinase (JNK)/c-Jun signaling pathway [47]. Vanicoside B has demonstrated significant anti-metastatic properties [43]. One proposed mechanism is its ability to counteract Cyclin-Dependent Kinase 8 (CDK8)-mediated signaling pathways, which are involved in tumor progression [43]. AS inhibits the P2RX7-mediated TGF-β/SMAD signaling pathway by enhancing PPARG expression, which in turn reduces EMT in TNBC [44]. AS-IV has been shown to inhibit both the proliferation and invasion of TNBC cells and to suppress tumor growth in animal models. This is achieved through mechanisms involving the downregulation of Vav Family Member 3 (Vav3) and the inactivation of the Rac1/MAPK signaling pathway [54]. SC reduces TNBC metastasis by targeting the tumor necrosis factor alpha (TNFα)/Tumor necrosis factor receptor 2 (TNFR2) pathway, which leads to the activation of runt-related transcription factor 1 (RUNX1) and increased production of granulocyte-colony stimulating factor (G-CSF) in TNBC-associated endothelial cells [52,53]. PD inhibits the migration, invasion, and adhesion of highly metastatic TNBC cells by downregulating matrix metalloproteinase-9 (MMP-9) and inhibiting epidermal growth factor receptor (EGFR), as well as the MAPK and PI3K/AKT pathways [49]. Ophiopogonin D has the potential to suppress TGF-β1-mediated metastatic behavior in TNBC cells by modulating the ITGB1/FAK/Src/AKT/β-catenin/MMP-9 signaling axis [55]. Rutin inhibits the migration and invasion of TNBC cells by reducing c-Met phosphorylation and blocking its downstream signaling pathways [51,56].

#### 2.2.2. Pro-Apoptosis

Studies have shown that glycoside compounds, such as icariin, Vanicoside B, Ginsenoside Rg3, Saikosaponin D, DLBS1425, Saxifragifolin A, and neohesperidin, can also induce apoptosis in TNBC cells through various mechanisms [43,47,57,58,59,60,61,62,63]. Icariin has been reported to induce apoptosis by causing an excessive accumulation of ROS in cells [47]. Vanicoside B induces apoptosis in TNBC cells through activation of the Skp2-p27 signaling pathway, achieved by inhibiting CDK8 activity [43]. Rg3 induces apoptosis in TNBC cells through the classical mitochondria-dependent caspase activation pathway. Additionally, Rg3 has been reported to induce apoptosis in MDA-MB-231 cells by blocking Nuclear Factor kappa B (NF-κB) signaling, inactivating ERK and AKT, and destabilizing mutant p53 [64,65]. SSD exhibits a strong anti-proliferative effect on TNBC cells by inducing apoptosis. Its potential molecular mechanism involves inhibiting the Wnt/β-catenin signaling pathway, rather than targeting EGFR and Neurotensin receptor 1 (NTSR1) [59]. Glycoside compounds have demonstrated potential as therapeutic agents for TNBC by inducing apoptosis through various mechanisms.

#### 2.2.3. Other Anti-TNBC Effects

In addition to inhibiting metastasis and inducing apoptosis in TNBC cells, glycoside compounds also exhibit other anti-TNBC activities, such as synergistic effects with other drugs, enhancing drug efficacy, and modulating the tumor microenvironment. Ginsenoside Rg1 enhances the cytotoxic effects of doxorubicin through a synergistic mechanism [66]. Additionally, Ginsenoside Rg1 has been shown to protect effectively against cisplatin-induced liver damage, reducing associated histological damage. This protective effect is mediated by inhibiting the Kelch-like epichlorohydrin-associated protein 1 (Keap1)-Nrf2 interaction, partly due to p62 accumulation, and primarily through increasing the production of Nrf2-related antioxidant proteins [67]. The combination of oxymatrine and AS-IV can enhance the immune system to combat TNBC by improving the tumor microenvironment and boosting the antitumor activity of T cells [45]. Anemoside A3 prevents the polarization of macrophages into the M2 type by decreasing the phosphorylation of Signal transducer and activator of transcription 3 (STAT3) protein, thereby suppressing M2 macrophage-driven tumor metastasis [68]. Glycoside compounds have shown potential in inhibiting TNBC. We can further explore additional candidate compounds with anti-TNBC activity.

Glycoside compounds inhibit the migration, invasion, and growth of TNBC cells by modulating various signaling pathways, such as MAPK, PI3K/AKT, CDK8, and ITGB1/FAK/Src/AKT. These compounds induce apoptosis in TNBC cells through multiple mechanisms: promoting excessive ROS accumulation, inhibiting CDK8 to activate the Skp2-p27 signaling pathway, triggering the mitochondria-dependent caspase activation pathway, blocking NF-κB signaling, inhibiting ERK and AKT, destabilizing mutant p53, and suppressing the Wnt/β-catenin signaling pathway. Furthermore, glycoside compounds may improve chemotherapy efficacy and reduce drug toxicity, offering better treatment options for immune-suppressed TNBC patients. However, the precise mechanisms are not yet fully understood, and need further investigation in order to enhance the use of glycoside compounds in treating TNBC.

**Table 2:** Profiles of glycosides in TCM with therapeutic effects on TNBC.

Natural Products	Structure	Sources	Concentration In Vitro	Dosage In Vivo	Biological Activities/Mechanisms	Potential Targets	Reference
Rutin	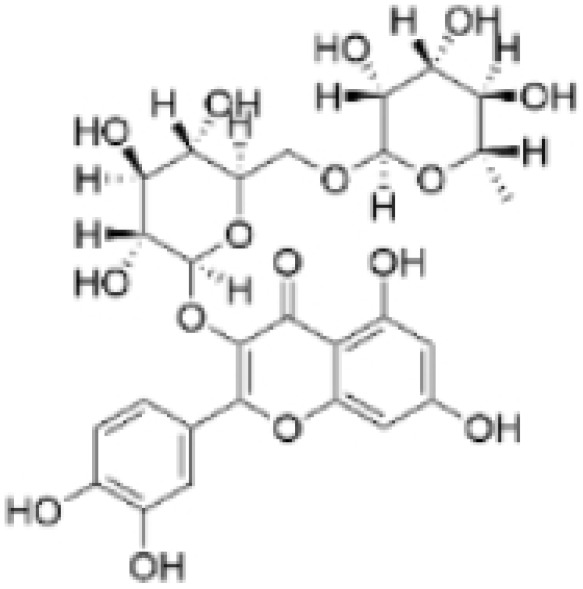	*Acacia erioloba* E. Mey.	150, 300 μM	30 mg/kg	Rutin acts as an inhibitor of c-Met Kinase and effectively inhibits the proliferation of TNBC cells both in vitro and in vivo.	/	[56]
Icariin	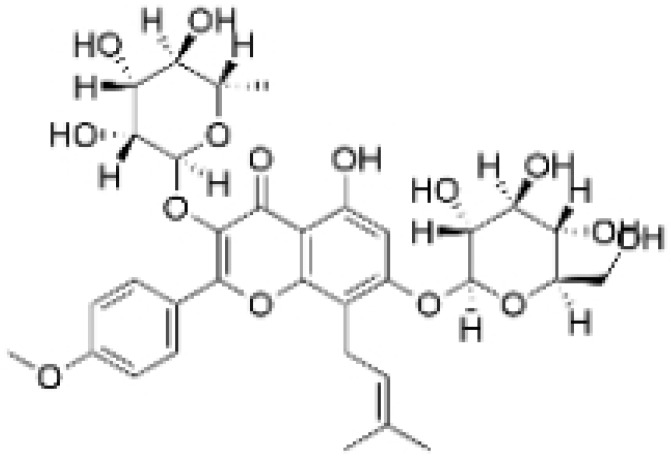	*Epimedium wushanense* Maxim.	5, 10, 20 μM	20, 40 mg/kg	Induces apoptosis and suppresses the migration of TNBC cells through modulation of the Sirtuin (Sirt) 6 /NF-κB signaling pathway.	/	[47]
Vanicoside B	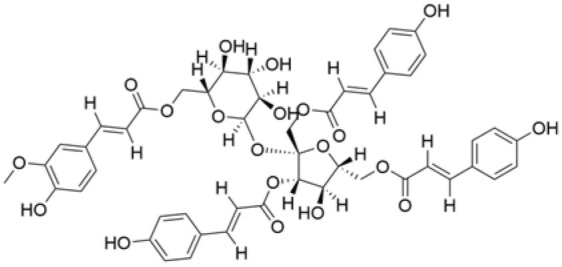	*Persicaria dissitiflora* H. Hara	5, 10, 20 μM	5, 10 mg/kg	The inhibition of CDK8-mediated signaling pathways leads to downregulation of EMT proteins and instigates cell cycle arrest and apoptosis in TNBC cells.	/	[43]
Anemoside A3	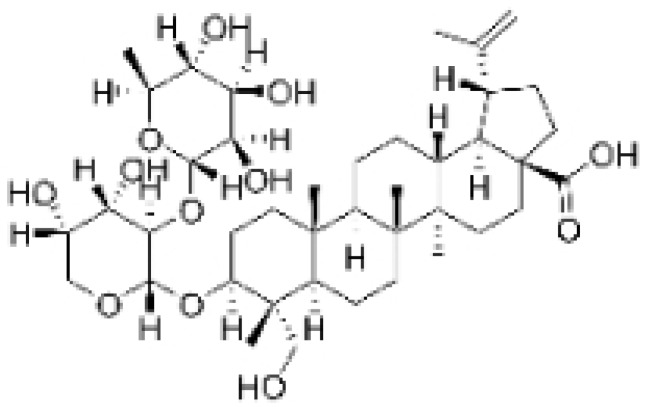	*Pulsatilla chinensis* (Maxim.) Klob.	50, 100 μg/mL	10, 20 mg/kg	Promotes differentiation of M0 macrophages into the M1 phenotype, which hinges upon the Toll-like receptor 4 (TLR4) /NF-κB/MAPK signaling cascade.	TLR4	[68]
Asiaticoside	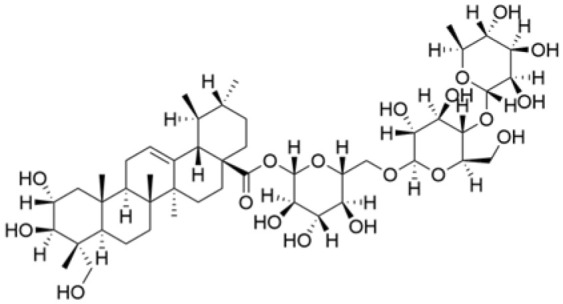	*Centella asiatica* (L.) Urban	0.25 μM	1, 2, 3 mg/kg	Inhibits P2RX7-mediated TGF-β/SMAD signaling through upregulation of PPARγ expression, thus attenuating EMT in TNBC.	PPARγ	[44]
Astragaloside IV	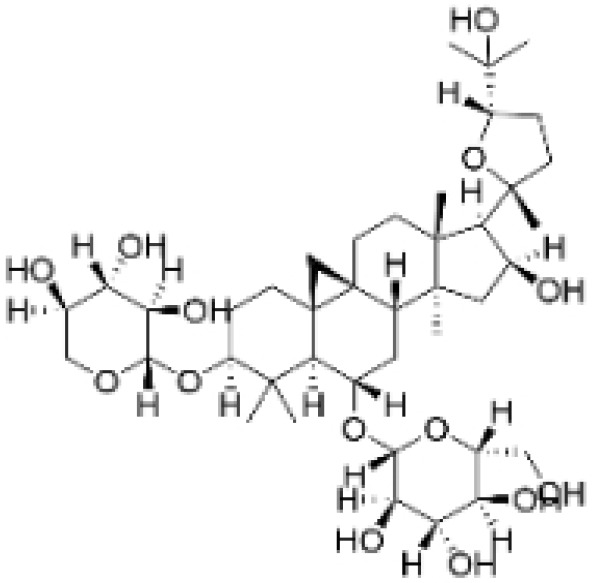	*Astragalus membranaceus* Bunge	20, 40 μg/mL	20, 40 mg/kg	Reduces Vav3 expression.	/	[54]
Ginsenoside Rg1	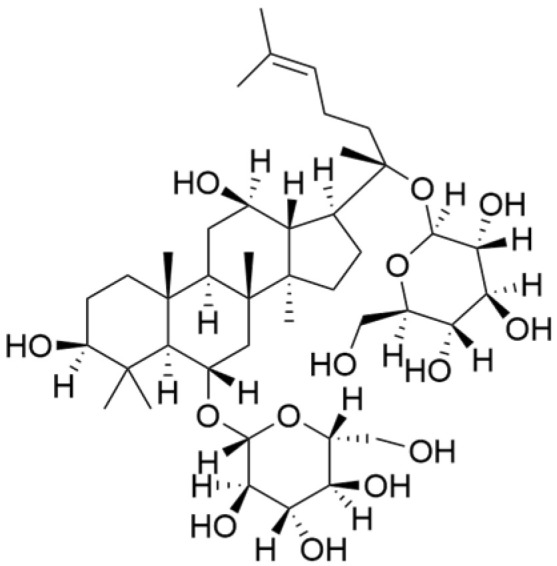	*Panax ginseng* C. A. Mey.	10 μM	/	Activates the DNA damage-response elements [Ataxia telangiectasia mutated protein kinase (ATM), H2A histone family member X (H2AX), Radiation-sensitive 51 (RAD51), X-ray repair cross-complementing protein 1(XRCC1), apoptosis-related genes (P21, TP53, Apoptotic peptidase activating factor 1 (APAF1), BAX, Caspase 3(CASP3), and Caspase 9(CASP9))] in MDA-MB-231 cells.	/	[66,69]
Ginsenosides Rg3	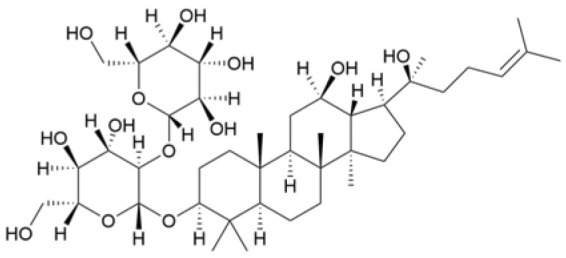	*Panax ginseng* C. A. Mey.	300 mg/mL	/	Increases the Bax/Bcl-2 ratio, causes mitochondrial membrane potential depolarization, leads to cytochrome c release, and induces caspase-3 and PARP cleavage.	/	[64,65]
Saikosaponin D	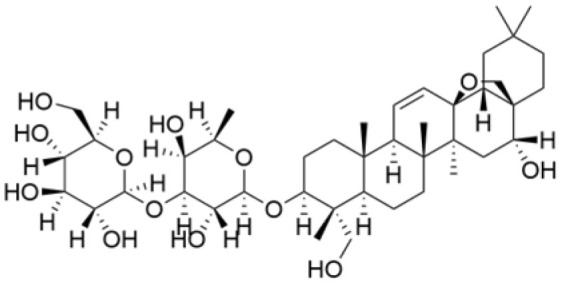	*Bupleurum rotundifolium* L.	10, 15, 30 μM	/	Suppresses the proliferation of TNBC cells through modulation of the β-catenin signaling cascade.	β-catenin	[70]
Scutellarin	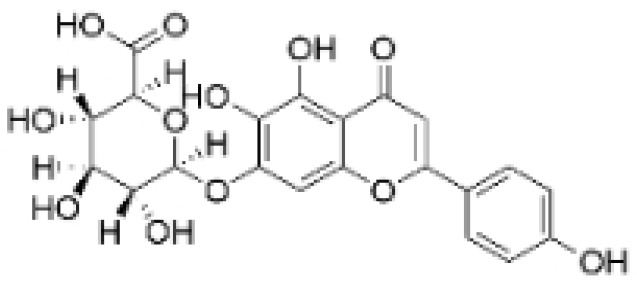	*Erigeron breviscapus* (Vaniot) Hand. -Mazz.	20 μM	10 mg/kg	Reduces TNBC metastasis through the targeting of TNFα/TNFR2-initiated activation of RUNX1 and subsequent production of G-CSF in TNBC-associated endothelial cells.	TNFα/TNFR2	[71]

### 2.3. Phenolics

**Figure 3:**
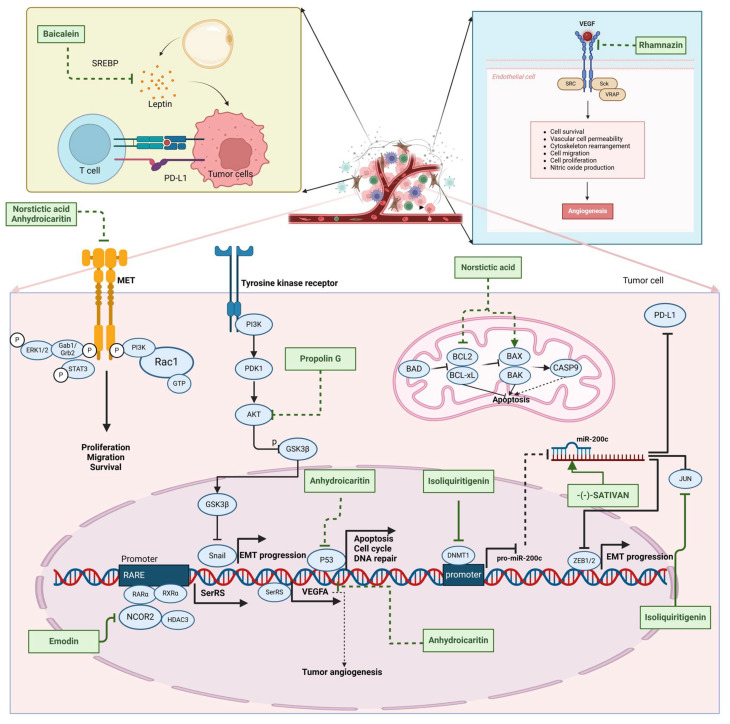
The regulation of phenolics in TNBC.

Phenolic compounds are composed of aromatic rings and hydroxyl groups. These compounds demonstrate a variety of biological activities, such as antioxidant, anti-inflammatory, antitumor, and antimicrobial effects [72,73,74,75]. Their antioxidant properties arise from the hydroxyl groups on the aromatic rings, which can donate electrons [75]. The natural world is rich in phenolic compounds, and recent studies have shown that some of these compounds can effectively combat TNBC (Figure 3, Table 3 and Table S1).

#### 2.3.1. Anti-Metastasis

Recent studies indicate that phenolic compounds can inhibit the metastasis of TNBC cells. For instance, (-)-sativan may regulate miR-200c, thereby affecting the EMT process and Programmed death-ligand 1 (PD-L1) expression in TNBC cells [76]. Similarly, isoliquiritigenin can also increase miR-200c levels, thereby suppressing the EMT process [77]. Propolin G inhibits the EMT in TNBC cells by degrading the cytoskeletal protein vimentin. This process is mediated by Glycogen synthase kinase 3β (GSK-3β) and regulated by Snail and Histone deacetylase 6 (HDAC6) [78]. Baicalein effectively reverses the expression of Interferon-induced protein with tetratricopeptide repeats 2 (IFIT2), which is associated with metastasis, recurrence, and poor prognosis in TNBC patients [79]. Norstictic acid significantly inhibits the proliferation, migration, and invasion of TNBC cells, while it shows minimal toxicity to non-tumorigenic MCF-10A mammary epithelial cells. Its potential mechanism involves the inhibition of c-Met phosphorylation [80]. Gigantol prevents TNBC cell migration and invasion by suppressing the Wnt/β-catenin signaling pathway [81].

#### 2.3.2. Anti-Angiogenesis

Phenolic compounds can inhibit the progression of TNBC by suppressing angiogenesis, primarily through the inhibition of VEGFA. Emodin exhibits anti-angiogenic effects in TNBC by targeting the transcriptional regulators nuclear receptor corepressor 2 (NCOR2) and Seryl-tRNA synthetase (SerRS), thereby suppressing VEGFA transcription [82]. Research shows that the hydroxyl group at the C-3 position of Emodin is crucial for its anticancer activity [83]. Rhamnazin, a new vascular endothelial growth factor receptor 2 (VEGFR2) inhibitor, blocks tumor angiogenesis and growth. Rhamnazin significantly reduced the proliferation, migration, and tube formation of human umbilical vascular endothelial cells (HUVECs) in vitro, and also inhibited sprout formation in rat aorta ring assays [84]. Cirsimaritin reduced angiogenesis in MDA-MB-231 cells by lowering the levels of vascular endothelial growth factor (VEGF), p-AKT, and p-ERK [85].

#### 2.3.3. Other Anti-TNBC Effects

Phenolic compounds not only inhibit angiogenesis and metastasis in TNBC, but also suppress its progression through other mechanisms. Anhydroicaritin induces apoptosis in TNBC cells [86]. Isoliquiritigenin inhibits the proliferation and migration of TNBC cells by reducing the expression of miR-374a, which negatively regulates PTEN and results in increased apoptosis and decreased invasiveness by inactivating the AKT pathway [77]. Kaempferol inhibits the expression of Sirt3 and Sirt6, which are potential therapeutic targets for TNBC due to their roles in cancer stem cell activity, chemotherapy resistance, and metastasis [87]. The combination of Morin and doxorubicin enhances the efficacy against TNBC [88]. Baicalein increases the sensitivity of TNBC cells to doxorubicin by promoting autophagy-mediated downregulation of CDK1 [89,90].

Recent studies have reported that phenolic compounds suppress metastasis and angiogenesis, induce apoptosis in TNBC cells, and enhance the sensitivity of TNBC to chemotherapy drugs. The inhibition of angiogenesis by phenolic compounds is closely linked to VEGFA, which plays a crucial role in this process by promoting the proliferation, migration, and lumen formation of endothelial cells, thereby directly influencing vascular growth and development. In the tumor microenvironment, VEGFA overexpression is strongly associated with tumor angiogenesis, creating conditions that support tumor growth [91,92]. Phenolic compounds affect TNBC cells through various mechanisms to prevent the onset and progression of the disease. For example, isoliquiritigenin has been shown to simultaneously suppress TNBC cell metastasis and induce apoptosis. Despite the promising results of phenolic compounds in anti-TNBC research, further studies are necessary to evaluate their potential for clinical application.

### 2.4. Alkaloids

**Figure 4:**
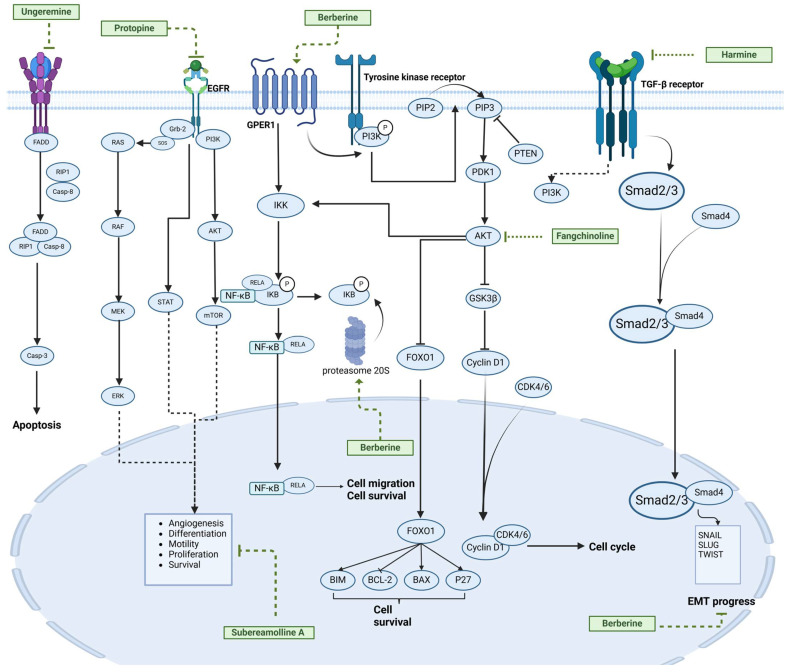
The regulation of alkaloids in TNBC.

Alkaloids are a group of naturally occurring compounds characterized by nitrogen-containing heterocyclic structures, and are frequently found in various plants. Current investigations indicate that alkaloids such as berberine, matrine, fangchinoline, and Dragmacidin D, sourced from diverse organisms, exhibit therapeutic promise against TNBC through the inhibition of proliferation [93,94,95,96,97,98] (Figure 4, Table 4 and Table S1).

**Table 3:** Profiles of phenolics in TCM with therapeutic effects on TNBC.

Natural Products	Structure	Sources	Concentration In Vitro	Dosage In Vivo	Biological Activities/Mechanisms	Potential Targets	Reference
(-)-Sativan(SA)	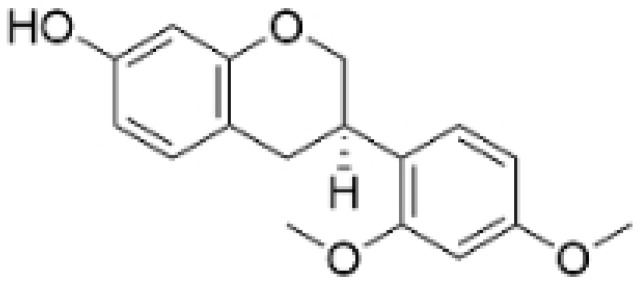	*Spatholobus suberectus* Dunn	5, 10, 20 μM	25, 50 mg/kg	Upregulates miR-200c to suppress the process of EMT.	/	[76]
Anhydroicaritin	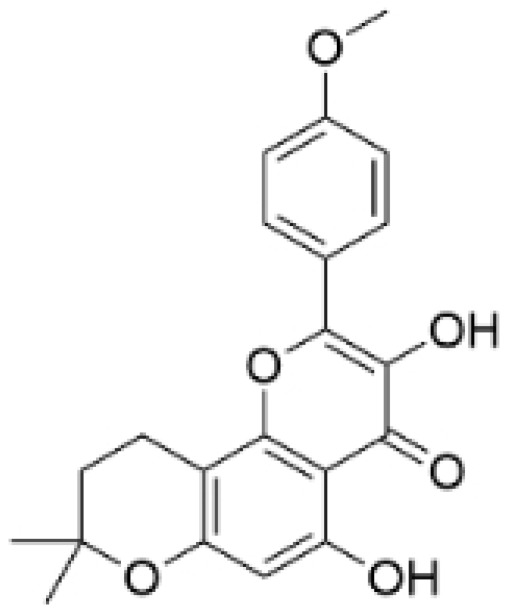	*Epimedium brevicornu* Maxim.	10, 20, 30 μM	20 mg/kg	Inhibits the HIF-1α/VEGFA signaling pathway.	/	[86]
Emodin	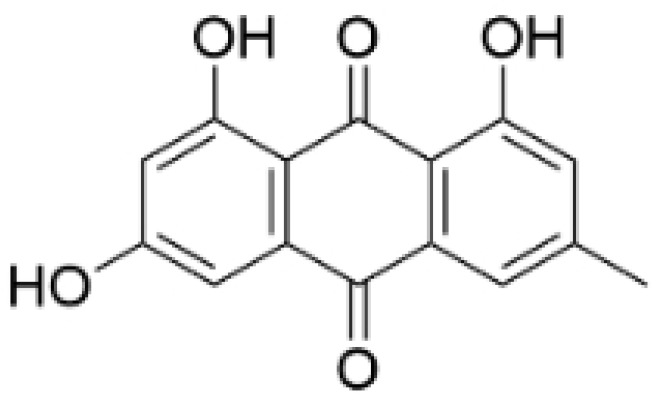	*Rheum palmatum* L.	0.25, 10, 25 μM	40 mg/kg	Regulates the activity of NCOR2; inhibits SIK3; suppresses TGF-β1.	NCOR2	[82]
Isoliquiritigenin	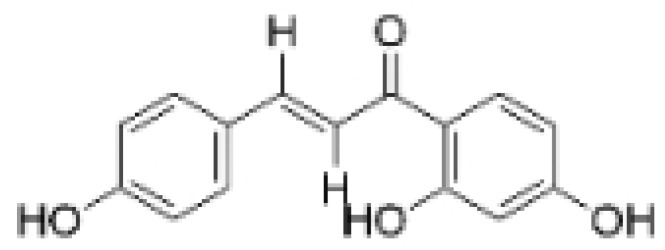	*Spatholobus suberectus* Dunn	10 μM	20, 40 mg/kg	Reduces miR-374a expression; elevates BAX protein and mRNA levels by inhibiting miR-374a; enhances miR-200c expression.	/	[99,100]
Kaempferol	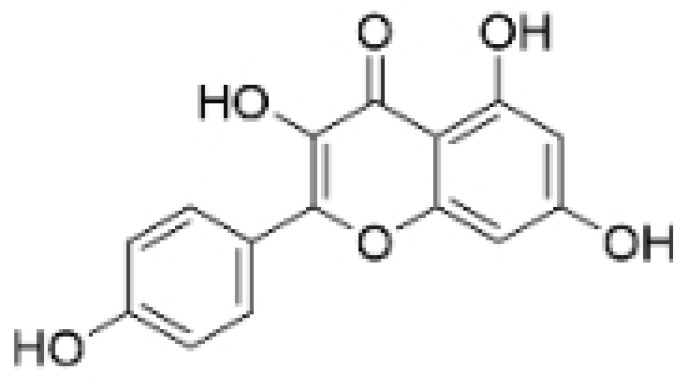	*Caragana frutex* (L.) C. Koch	12.5, 25 μg/mL,	/	Inhibits Sirt3 and Sirt6 proteins, inhibiting the PI3K/AKT pathway.	Sirts	[87]
Morin	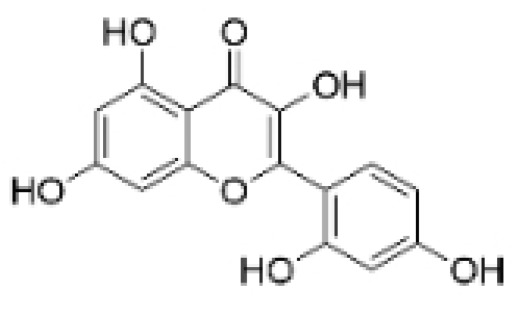	*Maclura pomifera* (Raf.) C. K. Schneid., *Petasites formosanus* Kitam.	10, 50, 100, 200 μM	10 mg/kg	Inhibits the growth and invasion of the highly metastatic BC cell line MDA-MB-231, which is partly achieved through the suppression of the AKT pathway.	/	[100,101]
Propolin G	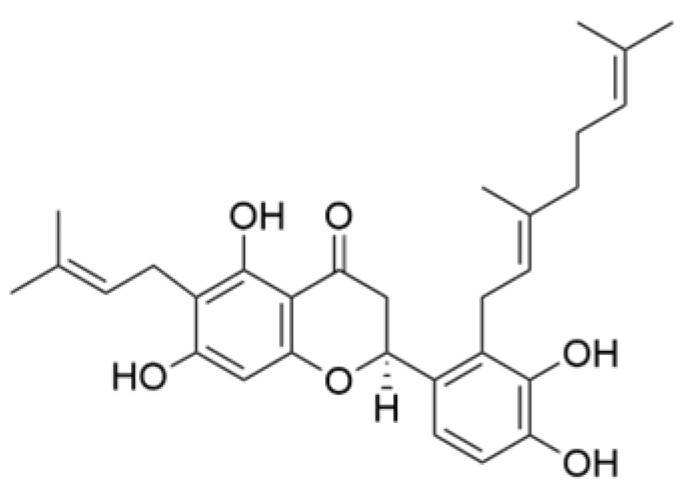	Taiwanese propolis	5, 10, 15, 20 μM	/	Inhibits the stabilization of vimentin protein, mediated by GSK-3β-Snail, and interferes with HDAC6, thereby impeding the suppression of cell migration and invasion in TNBC.	GSK-3β, HDAC6	[78]
Rhamnazin	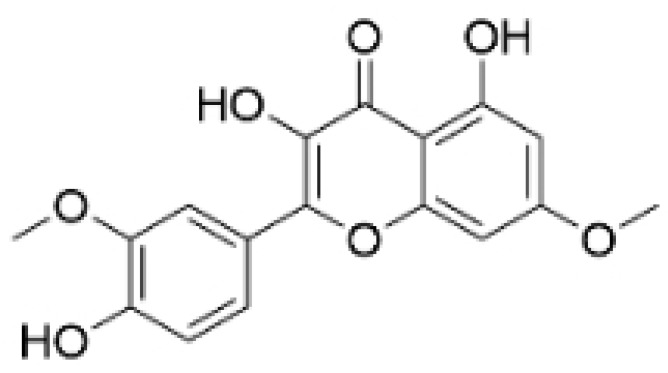	*Callicarpa kwangtungensis chun*, *Alocnemum strobilaceum*	10, 15, 20 μM	200 mg/kg	Suppresses VEGF-induced phosphorylation of VEGFR2 and its downstream signaling mediators in HUVECs.	VEGFR2	[84]
Baicalein	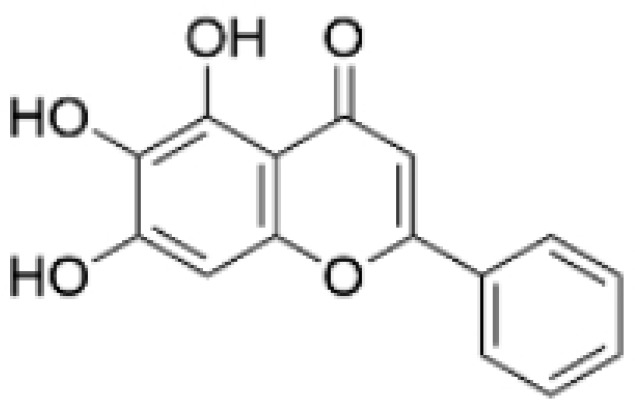	*Scutellaria baicalensis* Georgi	50, 100 ng/ml	50 mg/kg	Reverses the expression of IFIT2.	/	[89,102]
Norstictic acid	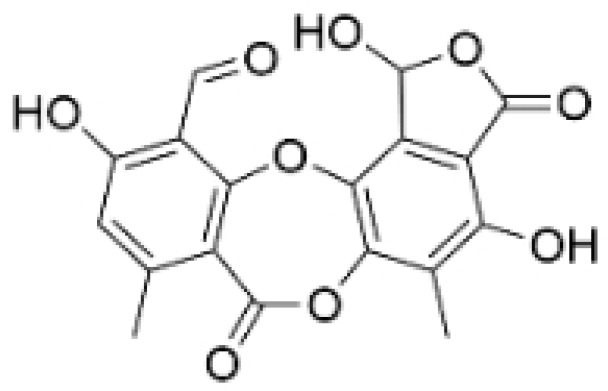	*Dimelaena oreina*, *Umbilicaria virginis*	15, 30 μM	15 mg/kg	Inhibits the proliferation, migration, and invasion of TNBC MDA-MB-231 cells.	/	[80]

#### Anti-Proliferation

Alkaloids have been reported to inhibit the proliferation of TNBC, with the PI3K/AKT pathway playing a crucial role. Fangchinoline inhibits the proliferation of MDA-MB-231 cells by targeting the AKT/GSK-3β/CCND1 signaling pathway. Additionally, fangchinoline has been shown to induce G1 phase cell cycle arrest and promote apoptosis in these cells [94]. Matrine disrupts the PI3K/AKT signaling pathway by reducing levels of PI3K, AKT, phosphorylated AKT, and phosphoglycerate kinase 1, leading to the inhibition of TNBC cell growth. Furthermore, matrine inhibits TNBC cell proliferation and induces apoptosis by suppressing HN1 expression [97]. Harmine inhibits the proliferation, invasion, and migration of MDA-MB-231 cells, while promoting apoptosis. This effect may result from the suppression of the EMT process and the PI3K/AKT signaling pathway [103]. Berberine inhibits TNBC cell growth by targeting the GPER1/NF-κB pathway, and also influences TNBC cell migration by reducing IL-6 secretion, and LH2 expression by modulating glycolysis [104,105]. Dragmacidin D effectively induces apoptosis in spheroids of TNBC [98]. Ungeremine exhibits cytotoxic effects on MDA-MB-231 cells by inducing ferroptosis, necroptotic apoptosis, and autophagy, alongside apoptosis, via caspase activation. It also affects mitochondrial membrane potential and increases ROS production [106].

In general, alkaloids inhibit cell migration, promote apoptosis, and block the cell cycle, thereby exerting antitumor effects in TNBC. Fangchinoline has been reported to inhibit TNBC cell growth, migration, and proliferation. Research indicates that substituting benzyl units in fangchinoline is crucial for significantly enhancing its antitumor activity [107]. This insight directs our efforts to synthesize fangchinoline derivatives, with the goal of finding more effective and less toxic candidates. Dragmacidin D effectively induces apoptosis in TNBC spheroids. However, it does not show cytotoxic effects on the same cell lines after prolonged incubation under traditional 2D growth conditions [98]. This suggests that establishing suitable in vitro models is essential. Most of these alkaloids have demonstrated anticancer activity in vitro, but lack data in vivo. Further in-depth research is needed.

While this review focuses on terpenoids, glycosides, phenolics, and alkaloids as the predominant anti-TNBC agents identified in our screening, a comprehensive list of additional compounds with reported activity (including the steroid, withanolide, unsaturated fatty acids, and furanocoumarins) is systematically cataloged in Supplementary Table S1, annotated with their chemical structures, source organisms, and experimental validation data.

**Table 4:** Profiles of alkaloids in TCM with therapeutic effects on TNBC.

Natural Products	Structure	Sources	Concentration In Vitro	Dosage In Vivo	Biological Activities/Mechanisms	Potential Targets	Reference
Fangchinoline	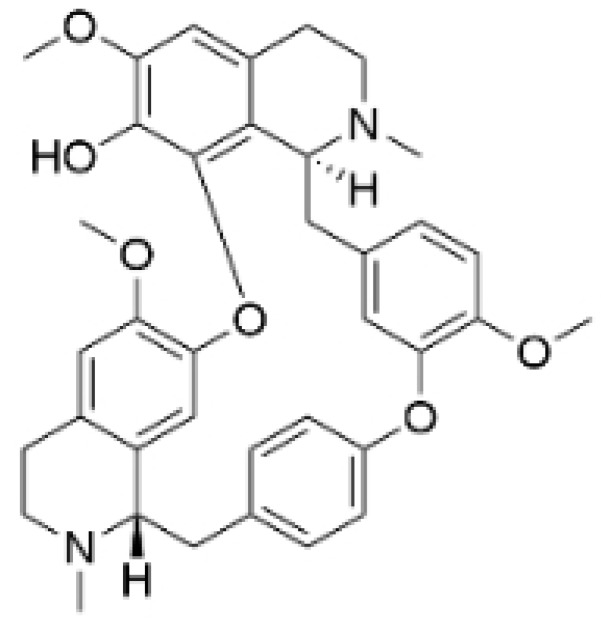	*Stephania tetrandra* (Radix)	6, 12, 24 μM5, 10, 30 μM	/	Inhibits the AKT/Gsk 3β/CCND1 signaling pathway; blocks cell cycle progression at the G1 phase; induces apoptosis.	/	[94,95]
Dragmacidin D	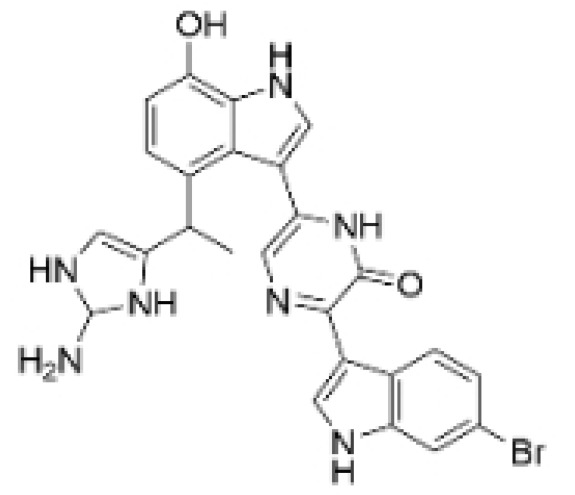	*Genus Spongosorites*	3.8, 7.6 μM	/	Induces apoptosis in TNBC spheroids.	/	[98]
Protopine	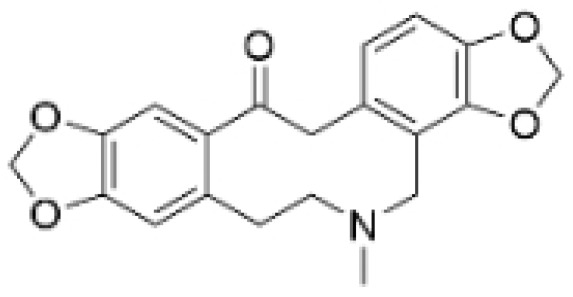	*Corydalis yanhusuo*	1, 10, 100 μM	/	Specifically inhibits the cell adhesion ability of MDA-MB-231.	/	[108]
Subereamolline A	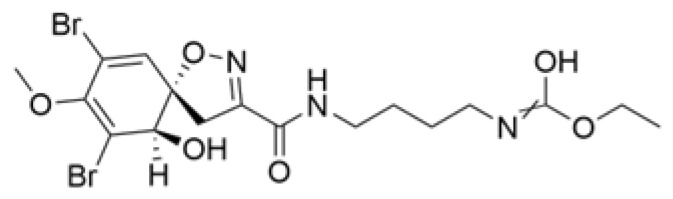	*Pseudoceratina arabica*	2 μM	/	Inhibits the migration and invasion of MDA-MB-231 cells.	/	[109]
Harmine	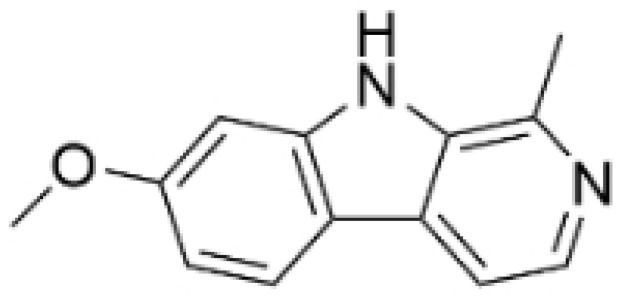	*Peganum harmala*	10 μM	/	Inhibits the process of EMT, associated with the PI3K/AKT signaling pathway.	/	[110]
Ungeremine	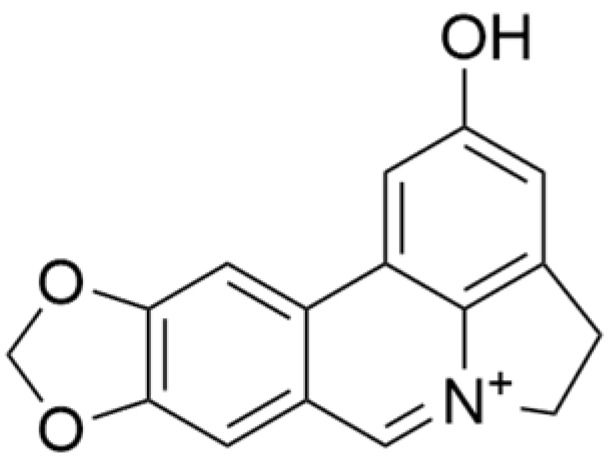	*Crinum zeylanicum*	3.67 μM	/	Induces ferroptosis, necroptosis, and autophagy, as well as apoptosis, mediated by caspase activation, MMP alteration, and increase ROS production.	/	[106]
Berberine	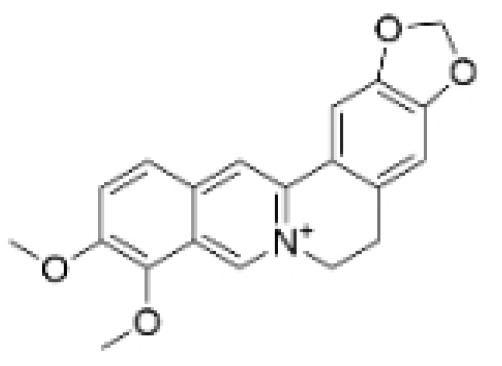	*Coptis chinensis*	1.25, 2.5, 5 μM	15, 30 mg/mL	Reduces IL-6 secretion and LH2 expression; modulates the GPER1/NF-κB pathway; inhibits the NLRP3 inflammasome pathway.	LH2	[107,111]
Matrine	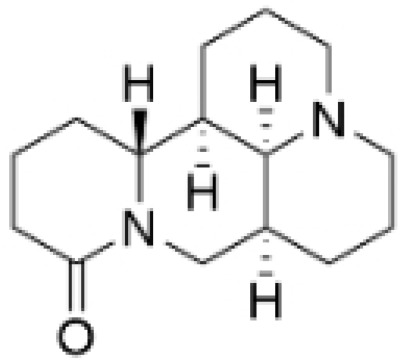	*Sophora flavescens*	2, 4 μM	/	Inhibits the PI3K/AKT signaling pathway; suppresses HN1 expression.	/	[93,97]

## 3. Natural Products: Mechanisms of Inhibiting TNBC

Emerging evidence highlights the multi-target mechanisms of NPs—including terpenes, glycosides, phenolics, and alkaloids—against TNBC. These compounds suppress tumor progression through diverse pathways, such as metastasis inhibition, apoptosis induction, cell cycle arrest, and tumor microenvironment modulation. Their polypharmacological effects often arise from modulating crosstalk between key signaling nodes, particularly the TGF-β, VEGFA, PI3K/AKT, Wnt/β-catenin, and MAPK pathways. By elucidating the interplay of these critical pathways, the following section further illuminates how NPs exert their therapeutic potential in TNBC.

### 3.1. TGF-β Signaling Pathway

Transcriptional regulation of EMT induced by TGF-β involves a complex cascade of events. Initially, in response to TGF-β, Smad2 and Smad3 become activated and form complexes with Smad4. These complexes then regulate the transcription of target genes by interacting with other DNA-binding transcription factors. During the induction of EMT, the activated Smads play a pivotal role in mediating transcriptional regulation through three distinct families of transcription factors. This process ultimately leads to the repression of epithelial marker gene expression and the activation of mesenchymal gene expression, which are hallmarks of EMT.

Additionally, TGF-β directly activates non-Smad signaling pathways. This activation occurs through the interaction of signaling mediators either directly with the TβRII and/or TβRI receptors, or via adaptor proteins. Among these non-Smad signaling responses, the activation of Erk MAP kinases, Rho family GTPases (Rho GTPases), and the PI3K /AKT pathway in response to TGF-β has been specifically linked to TGF-β-induced EMT. These pathways regulate various cellular processes, including cytoskeleton organization and cell growth, survival, migration, and invasion, which are crucial for the EMT process. The discovery of ITSN, AS, and harmine demonstrates their capacity to inhibit the TGF-β pathway, thereby suppressing EMT. This inhibition, in turn, contributes to the suppression of TNBC. Therefore, screening NPs that modulate the TGF-β pathway represents a promising strategy for the development of anti-TNBC therapies.

### 3.2. VEGFA Signaling Pathway

VEGFA is a key regulator of angiogenesis, stimulating endothelial cell proliferation, migration, and new blood vessel formation by its interaction with its primary receptors, VEGFR1 and VEGFR2 [112,113]. Tumor progression is frequently linked to enhanced angiogenesis, with VEGFA playing a central role in this process. As the tumor grows, increased VEGFA expression stimulates the formation of new blood vessels that provide essential nutrients to the tumor and support the spread of cancer cells [114,115]. Research indicates that elevated VEGFA levels are associated with poor clinical outcomes [116]. Compounds such as anhydroicaritin, rhamnazin, and emodin inhibit angiogenesis and metastasis by targeting the VEGFA pathway. VEGFA serves as a key target protein for treating TNBC, and developing and screening NPs that inhibit the VEGFA pathway could provide new therapeutic approaches for TNBC.

### 3.3. PI3K Signaling Pathway

The PI3K/AKT pathway is one of the most critical intracellular signaling pathways, playing a key role in various biological processes, such as cell growth, proliferation, metabolism, and survival. PI3K is a lipid kinase that activates downstream signaling by phosphorylating phosphatidylinositol 4,5-bisphosphate to form phosphatidylinositol 3,4,5-trisphosphate. The generation of PIP3 promotes the recruitment and activation of AKT, which regulates various downstream effectors, like mTOR, involved in processes like cell proliferation, survival, and metabolism [117]. Overactivation of the PI3K/AKT pathway is often associated with various cancer types. Studies have shown that abnormal activation of this pathway can result from mechanisms such as PIK3CA gene mutations or PTEN deletion or inactivation [118]. These genetic alterations lead to persistent activation of the pathway, promoting tumor cell proliferation and survival. Compounds such as AT-1, rhamnazin, norstictic acid, harmine, T-96, and matrine inhibit the PI3K/AKT pathway by targeting either PI3K or the phosphorylated forms of AKT. This inhibition induces apoptosis, suppresses cell migration, and reduces cell proliferation in TNBC. AKT promotes tumor cell survival by phosphorylating pro-apoptotic proteins, such as Bcl-2-associated death promoter and Caspase-9, thereby enhancing the cells’ ability to evade apoptosis, and it also plays a crucial role in tumor invasion and metastasis [119]. Overactivation of the PI3K/AKT pathway can enhance the motility and invasiveness of tumor cells, allowing them to breach the basement membrane and invade surrounding tissues [120]. Additionally, the PI3K/AKT pathway enhances the activation of tumor-associated fibroblasts, which supports tumor metastasis. Tumor growth and metastasis depend on an effective blood supply, and the PI3K/AKT pathway plays a crucial role in tumor angiogenesis. This pathway regulates the expression of VEGF, promoting the formation of new blood vessels [121]. Therefore, developing NP-based inhibitors of the PI3K/AKT pathway could provide new therapeutic strategies for TNBC.

### 3.4. Wnt/β-Catenin Signaling Pathway

The Wnt/β-catenin signaling pathway is essential for various physiological processes, including cell proliferation, differentiation, migration, and tissue homeostasis [122,123]. When Wnt proteins bind to Frizzled receptors on the cell surface, this pathway is activated, leading to the activation of β-catenin’s transcriptional activity. In the absence of Wnt signals, β-catenin is degraded in the cytoplasm. However, when Wnt signals are present, β-catenin accumulates and translocates to the nucleus, where it binds to T-cell factor/lymphoid enhancer factor to initiate the transcription of target genes [122].

Inhibiting the Wnt/β-catenin signaling pathway can effectively suppress tumor stem cell renewal, reduce cell proliferation, enhance sensitivity to chemotherapy, and inhibit tumor metastasis [123]. Saikosaponin D, a compound shown to significantly repress β-catenin and its downstream target genes, induces apoptosis in TNBC cells. Thus, developing NPs that target and inhibit the Wnt/β-catenin signaling pathway represents a promising strategy for treating TNBC.

### 3.5. MAPK Signaling Pathway

The MAPK signaling cascade involves three key kinases: MAPKKK, MAPKK, and MAPK, collectively known as mitogen-activated protein kinases. These pathways play a crucial role in the onset and progression of cancer [124]. Abnormal activation of MAPK pathways, often due to mutations in Ras proteins and rapidly accelerated fibrosarcoma proteins, leads to the continuous activation of the ERK1/2 pathway [125]. Research has shown that compounds such as EO, AT-1, Icariin, and AS-IV can inhibit the MAPK pathway, thereby suppressing TNBC.

NPs offer potential for targeting TNBC through the inhibition of other various critical pathways. For instance, kaempferol has been shown to suppress the expression of Sirt3 and Sirt6, which are associated with cancer stem cell functions, chemotherapy resistance, and metastasis. Another important target in TNBC therapy is Methionine (MET); research indicates that norstictic acid and rutin can effectively inhibit MET activity.

AC enhances the ubiquitination of GPX4, while britannin promotes the ubiquitination of ZEB1, a key transcription factor involved in EMT, thereby reducing TNBC cell metastasis. EGFR, through its interaction with ligands like epidermal growth factor (EGF), activates downstream signaling pathways that regulate cell proliferation, migration, and survival. Protopine has been identified as an inhibitor of EGFR expression, contributing to the suppression of TNBC. In addition, compounds such as ungeremine, Jatamanvaltrate P, ITSN, and AC can modulate autophagy, and consequently inhibit TNBC. Therefore, future research should delve deeper into the mechanisms of these pathways to discover natural compounds that can target these processes, offering new therapeutic avenues for TNBC treatment.

## 4. Challenges and Opportunities

TNBC, characterized by the absence of ER, PR, and HER2 immunostaining, is refractory to both endocrine therapy and conventional molecularly targeted therapies, making chemotherapy the primary systemic treatment approach. NPs are hailed as a primary source for drug discovery, due to their immense structural and physicochemical diversity.

Each type of compound that inhibits TNBC exhibits unique characteristics and commonalities. The antitumor effects of terpenoids encompass a broad spectrum of activities, including anti-proliferative, apoptotic, anti-angiogenic, and anti-metastatic actions. Glycosides primarily exert their anticancer effects through inhibiting metastasis, inducing apoptosis, and synergizing with other medications to enhance therapeutic efficacy and modulate the tumor microenvironment. Phenolic compounds manifest their anticancer effects mainly by inhibiting angiogenesis and metastasis in TNBC, and by increasing the cancer cells’ sensitivity to chemotherapeutic agents. Alkaloids predominantly achieve their anticancer effects through anti-proliferation. These effects are closely linked to their structural features. For example, the substitution of benzyl units in fangchinoline is a key pharmacophore that enhances its anti-proliferative effect on cancer cells. Meanwhile, research shows that the introduction of fluorine atoms or fluorinated groups significantly enhances the antitumor efficacy of the compound and prolongs its pharmacokinetic profile in vivo [107]. By systematically categorizing and organizing these NPs, we might infer the potential anticancer properties of compounds with similar structures, such as 3-aryl isocoumarins and 8-hydroxy-3-aryl isocoumarin, which are akin to kaempferol, morin, propolin G, rhamnazin, and baicalein, and may also exhibit certain anti-TNBC activities [126,127].

To comprehensively assess the therapeutic potential of NPs in TNBC, we compared their in vitro and in vivo efficacy with current standard treatments. NPs such as AC, AT-1, Vanicoside B, SA, and emodin demonstrated promising anticancer activity in cellular and animal models. For instance, AC exhibited potent effects against MDA-MB-231 cells (IC_50_ = 1.02 μM) and significantly inhibited tumor growth in murine TNBC models. Rutin reduced tumor volume by 53.85% and 65.72% in mouse TNBC models. Compounds like T-96, ITSN, and AS showed remarkable efficacy at low doses in vivo. While these findings are derived from preclinical studies, they provide critical scientific rationale for further development of NPs. Paclitaxel, a microtubule inhibitor, stabilizes microtubules to block cell cycle progression and induce apoptosis. Clinical trials have demonstrated its efficacy in TNBC, particularly when combined with carboplatin, improving overall and disease-free survival rates [128]. PARP inhibitors (e.g., Olaparib, Talazoparib) target BRCA-mutated TNBC by impairing DNA repair mechanisms. These agents are approved for metastatic TNBC patients with germline BRCA1/2 mutations [129]. NPs such as AC and T-96 have shown minimal toxicity in preclinical models, with no significant hepatorenal damage or adverse effects observed during prolonged administration. In contrast, paclitaxel is associated with dose-limiting neurotoxicity, myelosuppression, and hypersensitivity reactions, while PARP inhibitors may cause hematologic and gastrointestinal toxicities [130]. Although current research on NPs remains preclinical, advances in translational medicine are bridging the gap between laboratory discoveries and clinical applications. Further studies should prioritize evaluating their therapeutic potential in specific TNBC subtypes (e.g., BRCA-mutated tumors), for which NPs may offer distinct advantages in efficacy and tolerability.

NPs possess unparalleled structural diversity, a hallmark advantage derived from the vast biochemical repertoire of plants, animals, and microorganisms. These evolutionarily refined compounds exhibit intricate and unique architectures that far surpass the structural complexity of most synthetic counterparts. Such chemical distinctiveness enables NPs to engage with diverse biological targets through multifaceted interactions, positioning them as invaluable resources for drug discovery. In the context of TNBC—a malignancy characterized by dysregulation of multiple signaling pathways (e.g., PI3K/AKT/mTOR, VEGFA) and aberrant crosstalk within oncogenic networks—monotherapies targeting single pathways often yield suboptimal outcomes, due to compensatory resistance mechanisms. The polypharmacological properties of NPs enable them to simultaneously modulate multiple therapeutic targets, offering a promising strategy to address the heterogeneity and adaptive resistance of TNBC. For instance, AT-1 has been demonstrated to inhibit phosphorylation of the ERK family and suppress the expression of TPI1 and GPI, demonstrating significant anti-TNBC activity in preclinical models [21]. This multi-targeted approach enhances therapeutic efficacy and mitigates compensatory resistance mechanisms that are commonly observed in TNBC.

However, the clinical translation of NPs for TNBC faces multiple challenges, requiring prioritized research. First, there is an intricate relationship between the structural complexity of NPs and their toxicity; for example, the pentacyclic ring structure of TSN, which contains a carbonyl group, has been associated with marked hepatotoxicity, whereas ITSN, featuring an oxygen bridge, exhibits significantly reduced toxicity [37,131,132]. Second, conventional screening paradigms overemphasizing direct cytotoxicity (e.g., oxoflavidin’s prioritization based solely on MDA-MB-231 cell inhibition) risk overlooking multimodal therapeutic mechanisms, despite murine models remaining indispensable for preclinical validation [133]. Furthermore, critical gaps persist in understanding NP-associated safety profiles and comparative advantages over existing therapies. Future investigations must integrate structural optimization, mechanism elucidation, comprehensive safety assessments, and comparative efficacy studies to advance clinically viable NP-based TNBC therapeutics.

## 5. Conclusions

NPs exhibit unparalleled chemical diversity, underpinning their remarkable potential as reservoirs of bioactive compounds with multifaceted pharmacological properties. Emerging evidence highlights their growing significance in addressing TNBC, with numerous NPs demonstrating antitumor efficacy through diverse mechanisms.

In this review, we have compiled information on NPs with anti-TNBC properties. These natural agents predominantly comprise a variety of compounds, such as terpenoids, glycosides, phenolic substances, and alkaloids. A profound exploration has revealed their participation in crucial signaling pathways, specifically the TGF-β, VEGFA, PI3K/AKT, Wnt/β-catenin, and MAPK signaling pathways. Although these NPs exhibit potential anticancer activities, the studies on them remain at the preclinical basic research stage, and further exploration is still required.

## Data Availability

No new data were created or analyzed in this study. Data sharing is not applicable to this article.

## Supplementary Materials

The following supporting information can be downloaded at https://www.mdpi.com/article/10.3390/molecules30061201/s1. A comprehensive list of additional compounds with reported activity (including the steroid, withanolide, unsaturated fatty acids, and furanocoumarins) [134,135,136,137,138,139,140,141,142,143,144,145,146,147,148,149,150,151,152,153,154,155,156,157,158,159,160,161,162,163,164,165,166,167,168,169,170,171,172,173,174,175,176,177,178,179,180,181,182,183].

## Abbreviations

The following abbreviations are used in this manuscript:

TNBC	Triple-negative breast cancer
NPs	Natural products
BC	Breast cancer
WHO	World Health Organization
HER2	Human epidermal growth receptor 2
ER	Estrogen receptor
PR	Progesterone receptor
TCMs	Traditional Chinese medicines
HMs	Herbal medicines
FDA	Food and Drug Administration
AT-1	Atractylenolide-1
EMT	Epithelial–mesenchymal transition
AS	Asiaticoside
ITSN	Isotoosendanin
GPX4	Glutathione peroxidase 4
T-96	Demethylzeylasteral
AC	Anomanolide C
TPI1	Triosephosphate Isomerase 1
GPI	Glucose-6-phosphate isomerase
EO	Eupalinolide O
LSD1	Lysine-specific demethylase 1
PTEN	Phosphatase and tensin homolog
MA	Maackiain
ROS	Reactive oxygen species
CDK1	Cyclin-dependent kinase
CCND1	Cyclin D1
PARP	Poly adenosinediphosphate-ribose polymerase
BRCA	Breast cancer gene
TGF-β	Transforming growth factor-beta
VEGF	Vascular endothelial growth factor
PI3K	Phosphoinositide3kinase
AKT	Protein kinase B
Wnt	Wingless/Int-1
AS- IV	Astragaloside IV
SC	Scutellarin
PD	Platycodin D
TNFα	Tumor necrosis factor alpha
TNFR2	Tumor necrosis factor receptor 2
TGFβR1	TGF-β receptor type-1
ERK	Extracellular signal-regulated kinase
JNK	c-Jun N-terminal kinase
Rho GTPase	Rho family GTPases
NF-κB	Nuclear Factor kappa B
Bcl2	B-cell lymphoma 2
Bax	Bcl-2-associated X protein
c-Myc	Cellular myelocytomatosis oncogene
ZEB1	Zinc finger e-box binding homeobox 1
Cdc-2	Cell division cycle 2
MAPK	Mitogen-activated protein kinase
PTP1B	Protein tyrosine phosphatase 1B
CDK8	Cyclin-Dependent Kinase 8
Vav3	Vav Family Member 3
RUNX1	Runt-related transcription factor 1
G-CSF	Granulocyte-colony stimulating factor
MMP-9	Matrix metalloproteinase-9
EGFR	Epidermal growth factor receptor
NTSR1	Neurotensin receptor 1
Keap1	Kelch-like epichlorohydrin-associated protein 1
STAT3	Signal transducer and activator of transcription 3
Sirt	Sirtuin
TLR4	Toll-like receptor 4
ATM	Ataxia telangiectasia mutated protein kinase
H2AX	H2A histone family member X
RAD51	Radiation-sensitive 51
XRCC1	X-ray repair cross-complementing protein 1
APAF1	Apoptotic peptidase activating factor 1
CASP3	Caspase 3
CASP9	Caspase 9
PD-L1	Programmed death-ligand 1
miR	microRNA
GSK-3β	Glycogen synthase kinase 3β
HDAC6	Histone deacetylase 6
IFIT2	Interferon-induced protein with tetratricopeptide repeats 2
NCOR2	Nuclear receptor corepressor 2
SerRS	Seryl-tRNA synthetase
HUVECs	Human umbilical vascular endothelial cells
TSN	Toosendanin
MET	Methionine
VEGFR2	Vascular endothelial growth factor receptor 2
