# Supplementary material for: Natural Products as Novel Therapeutic Agents for Triple-Negative Breast Cancer: Current Evidence, Mechanisms, Challenges, and Opportunities
Source: Molecules. 2025 Mar 7;30(6):1201. doi: 10.3390/molecules30061201 (PMC11944566; doi:10.3390/molecules30061201)
Supplement: Supplementary file 1 [file molecules-30-01201-s001.zip › molecules-3473554-supplementary.pdf]

Natural Products as Novel Therapeutic Agents for Triple-Negative Breast Cancer: Current Evidence, Mechanisms, Challenges and Opportunities

Qingzhou Li [a,1], Zhen Ye [b,1], Guilin Wang [b], Yuhui Chen[b], Jinghong Deng[b], Dong Wang [a,b,*], Yumei Wang [a,b,**]

[a] State Key Laboratory of Southwestern Chinese Medicine Resources, School of Pharmacy, Chengdu University of Traditional Chinese Medicine, Chengdu, Sichuan, China.

[b] School of Basic Medical Sciences, Chengdu University of Traditional Chinese Medicine, Chengdu, Sichuan, China.

[*] Correspondence to: Dong Wang, School of Basic Medical Sciences, Chengdu University of Traditional Chinese Medicine, Chengdu, 610000, Sichuan, China.

[**] Correspondence to: Yumei Wang, School of Basic Medical Sciences, Chengdu University of Traditional Chinese Medicine, Chengdu, 610000, Sichuan, China.

E-mail addresses: dwang@cdutcm.edu.cn [Dong Wang], yumeiwang@cdutcm.edu.cn [Yumei Wang].

[1] These authors contributed equally to this work.

Supplementary Table S1

Natural products	Type of structure	Sources	Effective dosages	Mechanisms	References
Cupresins A	Terpenes	Cupressus macrocarpa	19.9 [μ g/mL]	/	[132]
Cupresins B	Terpenes	Cupressus macrocarpa	14.8 μM	/	[132]
Alpha-Santalol	Terpenes	Magnolia officinalis and Santalum album	20, and 40 μM	Inhibition of β-catenin phosphorylation.	[133]
Oleanolic acid	Terpenes	Wrightia tomentosa Roem. & Schult.	4.4 μM	The combination use of these drugs can promote apoptosis in TNBC cells.	[134]
Ursolic acid	Terpenes	Wrightia tomentosa Roem. & Schult.			
Hibisceusone A	Terpenes	Hibiscus tiliaceus	8.89 ± 0.64 μM	/	[135]

Hibisceusone B	Terpenes	Hibiscus tiliaceus	3.12 ± 0.58 μM	/	[135]
Hibisceusone C	Terpenes	Hibiscus tiliaceus	8.89 ± 0.64 μM	/	[135]
Saponin D Rhamnose β-hederin [DRβ-H]	Terpenes	Clematis ganpiniana	20 to 80 μg/mL	Inhibited cell migration and invasion under non-cytotoxic concentrations.	[136]
Alantolactone	Terpenes	Inula helenium L.	2.5, 5, and 10 μM	Suppressed the activity of STAT3.	[137]
Isoalantolactone	Terpenes	Inula helenium L.	2.5, 5, and 10 μM	Suppressed the expression of p-STAT3 [Y705].	[138]
Igalan	Terpenes	Inula helenium L.	2.5, 5, and 10 μM	Suppressed the expression of p-STAT3 [Y705].	[138]
Dugesialactone	Terpenes	Inula helenium L.	2.5, 5, and 10 μM	Suppressed the expression of p-STAT3 [Y705].	[138]
Alloantolactone	Terpenes	Inula helenium L.	2.5, 5, and 10 μM	Suppressed the expression of p-STAT3 [Y705].	[138]
Curcumin	Terpenes	Curcuma longa	30 to 100 μM	Suppressed TLR4/TRIF/IRF3 signaling pathway.	[139]
Parthenolide	Terpenes	Tanacetum parthenium	10 to 100 μM	/	[140]
Cucurbitacin B	Terpenes	Trichosanthes cucumerina L	1 to 100 μM	Interfered with the polymerization of microtubules and induced the translocation of nucleophosmin/B23, ultimately leading to cell cycle arrest at the G2/M phase and the commencement of apoptosis.	[33]
Eriocalyxin B	Terpenes	Isodon eriocalyx var. laxiflora	0.49 ± 0.01 μM IC50 [48h]]	Modulated the EGFR/MEK/ERK signaling cascade.	[141]
7β-[3-Ethyl-cis-crotonoyloxy]-1α-[2-	Terpenes	Tussilago farfara L. [Farfarae Flos]	2.5 to 10 μM	Suppression of the JAK/STAT3 pathway induces apoptosis in TNBC	[142]

methylbutyryloxy]-3,14-dehydro-Z-notonipetranone [ECN]				cells.	
Canusesnol K	Terpenes	Pterocarpus santalinus L. f.	35.07 ± 4.9 μM	/	[142]
Ganoderic acid Y	Terpenes	Ganoderma theaecolum	49.1 μM	/	[143]
7-oxo-ganoderic acid Z	Terpenes	Ganoderma theaecolum	75.8 μM	/	[143]
Pomolic acid [PA]	Terpenes	Euscaphis japonica	10 μM	PA suppressed EGF-induced HIF1α protein translation by inhibiting the p38-MAPK and mTOR kinase signaling pathways and played a novel anti-angiogenic role.	[144]
3β-trans-cinnamoyloxy-2α-hydroxy-urs-12-en-28-oic acid [CHUA]	Terpenes	Malus pumila Mill.	1 to 32 μM	CHUA induced apoptosis in MDA-MB-231 cells through mitochondrial pathways, with certain proteases distinct from caspase-3 potentially accounting for the degradation of PARP.	[145]
Leariifolianoid E	Terpenes	Inula leariifolia Turcz	1 to 5 μM	Arrested cell cycle.	[30]
3β-hydroxylup-20[29]-ene-27,28-dioic acid dimethyl ester	Terpenes	Plumbago zeylanica L.	1 to 500 μM	Inhibited metastasis.	[146]
Cycloart-23E-ene-3β, 25-diol	Terpenes	Euphorbia macrostegia	58.19 ± 12.3 μM	Upregulated the UPR signaling pathway	[29]
3-o-cispcoumaroyl alphitolic acid	Terpenes	Ziziphus jujuba	11.5 ± 1.8 μM	/	[147]
3-o-transpcoumaroyl alphitolic acid	Terpenes	Ziziphus jujuba	10.5 ± 1.1 μM	/	[147]

2-o-transpcoumaroyl alphitolic acid	Terpenes	Ziziphus jujuba	11.4 ± 1.3 μM	/	[147]
2-o-cispcoumaroyl alphitolic acid	Terpenes	Ziziphus jujuba	12.5 ± 2.2 μM	/	[147]
Germacrone	Terpenes	Rhizoma curcuma	25 to 400 μM	Induced apoptosis and arrested cell cycle	[32]
Cryptotanshinone	Terpenes	Salvia miltiorrhiza	20 μM	Blocked TH Blocked the THEMIS2–P-MET axis in TNBC; reprogrammed M2 macrophages and tumor-associated macrophages [TAMs] into an M1 phenotype.	[148, 149]
β-Bisabolene	Terpenes	Commiphora guidottii	100, and 200 μg/mL	Induced apoptosis	[27]
Taraxerol	Terpenes	Taraxacum mongolicum	40, 80, and 120 μM	Inhibited the migration and invasion of MDA-MB-231 cells through the ERK/Slug axis.	[150]
Dihydrotanshinone-I[DHTS]	Terpenes	Salvia miltiorrhiza	2 μM	Inhibited the migration of highly aggressive TNBC cells by obstructing EMT.	[151]
Dioscin	Glycosides	Dioscorea villosa	6.7 μM	Enhanced the expression of GATA-binding protein 3 [GATA3] through the modulation of both transcriptional and translational levels.	[152]
Amygdalin	Glycosides	Prunus armeniaca [apricot] and Prunus persica [peach].	69.9 ± 1.52 mM	The combined use of cisplatin and pemetrexed achieves synergistic effects while reducing toxicity.	[153]
Platycodin D	Glycosides	Platycodon grandiflorum	5, 15, and 20 μM	Inhibited the PI3K/Akt and MAPK pathways, as well as suppressed NF-κB activity.	[49]
Neohesperidin	Glycosides	Poncirus trifoliata	32.5 ± 1.8 μM	Induced apoptosis.	[60]

Diosgenin	Glycosides	Allium cernuum, and Dioscorea hispida	5, 10, and 20 µM	Suppressed Vav2 activity.	[154]
Aspiletreins A-C [1-3]	Glycosides	Aspidistra letreae	7.75 ± 0.74 µM<br>20.46 ± 3.1 µM<br>19.09 ± 1.01 µM	Inhibited cell proliferation.	[155]
Saxifragifolin A	Glycosides	Androsace umbellata	3.88 µg/mL	Induced apoptosis in MDA-MB-231 cells through ROS-mediated and caspase-dependent pathways.	[63]
20[S]-protopanaxadiol [PPD]	Glycosides	Panax ginseng C. A. Mey.	5.87 µM	Induced caspase-dependent apoptosis	[156]
Uzarigenin [1]	Glycosides	Calotropis gigantea	> 30 µM	2.1 ± 0.3 [EC50 [µM] ± SEM [EC50 Values of Cardenolides for Na+/K+ ATPase Inhibition]]	[157]
Coroglaucigenin [2]	Glycosides	Calotropis gigantea	20 ± 4 µM	1.1 ± 0.3 [EC50 [µM] ± SEM [EC50 Values of Cardenolides for Na+/K+ ATPase Inhibition]]	[157]
Desglucouzarin [3]	Glycosides	Calotropis gigantea	10 ± 1 µM	1.1 ± 0.3 [EC50 [µM] ± SEM [EC50 Values of Cardenolides for Na+/K+ ATPase Inhibition]]	[157]
Frugoside [4]	Glycosides	Calotropis gigantea	1.25 ± 0.09 µM	0.50 ± 0.08 [EC50 [µM] ± SEM [EC50 Values of Cardenolides for Na+/K+ ATPase Inhibition]]	[157]
Frugosidal [5]	Glycosides	Calotropis gigantea	10 ± 2 µM	2.5 ± 0.2 [EC50 [µM] ± SEM [EC50 Values of Cardenolides for Na+/K+ ATPase Inhibition]]	[157]
Glucofrugoside [6]	Glycosides	Calotropis gigantea	0.98 ± 0.06 µM	0.6 ± 0.2 [EC50 [µM] ± SEM [EC50 Values of Cardenolides for Na+/K+ ATPase Inhibition]]	[157]
Uscharin [7]	Glycosides	Calotropis gigantea	0.102 ± 0.004 µM	0.91 ± 0.03 [EC50 [µM] ± SEM [EC50 Values of Cardenolides for Na+/K+ ATPase Inhibition]]	[157]
Calotoxin [8]	Glycosides	Calotropis gigantea	0.63 ± 0.04 µM	0.30 ± 0.06 [EC50 [µM] ± SEM [EC50 Values of Cardenolides for Na+/K+	[157]

				ATPase Inhibition]]	
Calotropin [9]	Glycosides	Calotropis gigantea	0.44 ± 0.08 μM	0.36 ± 0.04 [EC50 [μM] ± SEM [EC50 Values of Cardenolides for Na+/K+ ATPase Inhibition]]	[157]
DLBS1425	Glycosides	Phaleria macrocarpa	5, 25, and 50 μg/mL	Induced apoptosis of MDA-MB-231 cells and inhibiting proliferation, regulated the PI3-K/AKT pathway.	[61]
β-Sitosterol-d-glucoside [β-SDG]	Glycosides	Ipomoea batatas [L.] Lam.	64 μM	/	[158]
Ophiopogonin D	Glycosides	Ophiopogon japonicus	10.52 μM	Suppressed TGF-β1-mediated metastatic behavior of MDA-MB-231 cells by regulating ITGB1/FAK/Src/AKT/β-catenin/MMP-9 signaling axis	[55]
Plantamajoside	Glycosides	Herba Plantaginis	31.25 to 500 μg/mL	Inhibited matrix metalloproteinase-9 and -2 activity	[159]
Gypensapogenin I	Glycosides	Gynostemma pentaphyllum [Thunb.] Makino	5, 10, and 20 μM	The inhibition of AKT/GSK3β/β-Catenin and Notch-1 signaling pathways.	[160]
1,3-Dicaffeoylquinic acid（1,3-DCQA）	Phenolics	Mikania hirsutissima, and Aster indicus	81.85 ± 3.56 μM	Targeted 14-3-3 tau inhibits the proliferation and metastasis of human breast cancer cells via the IL6/JAK2/PI3K pathway.	[161]
Cirsimaritin	Phenolics	Cirsium japonicum var. maackii	3.125 to 200 μM	Inhibited metastasis.	[85]
Gigantol	Phenolics	Several medicinal orchids	50, 75, and 100 μM	Inhibited TNBC cells' migration and invasion while also being able to suppress the Wnt/β-catenin signaling pathway.	[81]
Samarones A	Phenolics	Syzygium samarangense	25.57 ± 2.16 μM	/	[162]

Samarones B	Phenolics	Syzygium samarangense	27.57 ± 4.76 μM	/	[162]
Samarones C	Phenolics	Syzygium samarangense	28.26 ± 4.52 μM	/	[162]
Jambones E	Phenolics	Syzygium samarangense	28.26 ± 3.15 μM	/	[162]
Jambones F	Phenolics	Syzygium samarangense	12.01 ± 1.31 μM	/	[162]
Jambones G	Phenolics	Syzygium samarangense	4.02 ± 0.87 μM	/	[162]
Jamunone B	Phenolics	Syzygium samarangense	37.83 ± 3.42 μM	/	[162]
2-pentadecyl-5,7-didydroxy-chromone	Phenolics	Syzygium samarangense	7.196 ± 1.75 μM	/	[162]
Oxoflavidin	Phenolics	Coelogyne fuscescens Lindl. var. brunnea	26.26 ± 4.33 μM	Induced apoptosis	[162]
Oxyresveratrol	Phenolics	Maclura pomifera, Gnetum montanum	40 μM	Induced ROS-mediated apoptotic-like cellular demise, involving mitochondrial membrane depolarization, AIF translocation to the nucleus, and DNA fragmentation, ultimately leading to caspase-independent cell death in MDA-MB-231 cells.	[163]
Chrysin	Phenolics	Chinese propolis [CP]	10, 20, and 40 μM	HDAC8 enzymatic activity [EC [50] = 40.2 Mm]	[164]
Erythraddison II	Phenolics	Erythrina addisoniae [Leguminosae]	4.57 ± 0.60 μM	Protein tyrosine phosphatase 1B [PTP1B] inhibitors	[165]
Erythraddison III	Phenolics	Erythrina addisoniae [Leguminosae]	6.92 ± 0.27 μM	Protein tyrosine phosphatase 1B [PTP2B] inhibitors	[165]

Erythraddison IV	Phenolics	Erythrina addisoniae [Leguminosae]	6.34 ± 0.40 μM	Protein tyrosine phosphatase 1B [PTP3B] inhibitors	[165]
Echrenoneb10	Phenolics	Erythrina addisoniae [Leguminosae]	3.97 ± 0.17 μM	Protein tyrosine phosphatase 1B [PTP4B] inhibitors	[165]
Cinnamoyl-phloroglucinols, xanchryones C	Phenolics	Xanthostemon chrysanthus	25.26 ± 0.35 μM	/	[166]
Resveratrol	Phenolics	Red grapes	5, and 15 μM	Inhibited the migration of TNBC cells by suppressing the expression of MED28 and the EGF-mediated signaling pathway.	[167]
Cyanidin-3-O-sambubioside	Phenolics	Fruits of Acanthopanax sessiliflorus	1, 3, 10, and 30 μg/mL	Inhibited the migration of MDA-MB-231 cells by downregulating MMP-9 expression.	[168]
Proanthocyanidins	Phenolics	Lathyrus laxiflorus, Vitis amurensis	20, and 40 μg/mL	Induced apoptosis.	[169]
Calcitrinone A	Phenolics	Callistemon citrinus	7.5 ± 0.2 μM	Reduced intracellular ATP levels in MDA-MB-231 cells, impedes proliferation, and promotes apoptosis.	[170]
2-methyl-1,3,6-trihydroxy-9,10-anthraquinone-3-O-[6′-O-acetyl]-α-rhamnosyl [1 → 2]-β-glucoside	Phenolics	Rubia philippinensis	49.44 ± 0.78 μM	/	[171]
2-methyl-1,3,6-trihydroxy-9,10-anthraquinone	Phenolics	Rubia philippinensis	59.22 ± 0.40 μM	/	[171]
Alizarin	Phenolics	Rubia philippinensis	48.64 ± 0.33 μM	/	[171]
Yielded xanthopurpurin	Phenolics	Rubia philippinensis	14.65 ± 1.45 μM	/	[171]
Lucidin-ω-methyl ether	Phenolics	Rubia philippinensis	13.03 ± 0.33 μM	/	[171]

Carvacrol	Phenolics	Callistemon citrinus, and Perilla frutescens	100 μM	/	[172]
Isoliquiritigenin	Phenolics	Spatholobus suberectus Dunn	8.696 μM	Downregulated miR-374a expression	[93]
Erucin	Isothiocyanate	Diplotaxis tenuifolia [wild rocket] and Eruca sativa [arugula, or cultivated rocket]	30 μM	Restricted proliferation, migration, and invasion.	[173]
[22E,24R]-5a,8a-epid ioxyergosta-6,22-dien-3b-ol	Steroid	Solanum violaceum	16.74±0.33 μg/mL	/	[174]
[22E,24R]-5a,8a-epidioxyergo sta-6,9[11],22-trien-3b-ol	Steroid	Solanum violaceum	10.37±0.72 μg/mL	/	[174]
Ergosterol	Steroid	Amauroderma rude	10, and 30 μg/mL	Inhibited migration and invasion	[175]
Physapruin A [PHA]	Withanolide	Physalis L.	1, 2.5, 5, and 10 μM	Induced endoplasmic reticulum strain via oxidative strain	[176]
4β-hydroxywithanolide E	Withanolide	Physalis peruviana [golden berry]	0.5, 1, 2, 5, and 10 μM	Inhibited the proliferation of TNBC cells primarily through suppression of the Akt signaling pathway.	[177]
Tubocapsanolide A	Withanolide	T. anomalum.	1.89 ± 1.03 μM	Induced apoptosis.	[178]
Withaferin A	Withanolide	Iochroma gesnerioides, Withania coagulans	4 μM	Induced ROS release, leading to endoplasmic reticulum stress and subsequent cytoplasmic vacuolation-mediated cell death in MDA-MB-231 cells.	[179]
Punicic acid	Unsaturated fatty acids	Punica granatum	40 μM	Induced apoptosis in cells also led to disruption of mitochondrial membrane potential.	[180]
Furospinulosin 1	Furanocoumarins	Hippospongia, Ircinia, Spongia, Fasciospongia, Idia, and Smenospongia	20 to 0.156 μg/mL	/	[181]

Goniothalamin [GTN]	Steroidal lactone	Genus Goniothalamus	20, 40, and 80 μM	Inhibited the EGFR/FAK/Src and EMT signaling pathway.	[182]
Amorfrutin A	Stilbenoid	Amorpha fruticosa L.	20 μM	Inhibited the expression of p-STAT3 [Tyr705]	[183]
